# Berberine as a Multifunctional Adjuvant in Cancer Therapy: Mechanistic Insights, Nanotechnological Strategies, and Translational Challenges

**DOI:** 10.3390/ph19040613

**Published:** 2026-04-13

**Authors:** Yıldız Özalp, Tarek Alloush, Nedime Serakıncı, Murat Kartal

**Affiliations:** 1 Department of Pharmaceutical Technology, Faculty of Pharmacy, Istinye University, Istanbul 34460, Türkiye; 2 Institute of Health Sciences, Istanbul University, Istanbul 34126, Türkiye; tarek.alloush@ogr.iu.edu.tr; 3 Department of Pharmaceutical Technology, Faculty of Pharmacy, Istanbul University, Istanbul 34126, Türkiye; 4 Department of Molecular Biology and Genetics, Faculty of Arts and Sciences, Cyprus International University, Nicosia 99258, Cyprus; nserakinci@ciu.edu.tr; 5 Department of Pharmacognosy, Faculty of Pharmacy, Bezmialem Vakıf University, Istanbul 34093, Türkiye; mkartal@bezmialem.edu.tr

**Keywords:** berberine, multidrug resistance, nanotechnology, bioavailability enhancement, cancer therapy

## Abstract

Multidrug resistance (MDR) and chemotherapy-associated toxicity remain major challenges limiting the success of cancer treatments. In this context, berberine (BBR), an isoquinoline derivative belonging to the barberry family, has emerged as a promising adjuvant that can enhance the efficacy of chemotherapy while potentially mitigating its side effects. The findings indicate that berberine enhances the therapeutic effect of several drugs, such as doxorubicin, cisplatin, tamoxifen, and 5-fluorouracil, through multiple mechanisms including the inhibition of ABC transporters, regulation of autophagy, and synergistic enhancement of reactive oxygen species generation. Advanced pharmaceutical and nanotechnological formulations, including cyclodextrin complexes, solid dispersions, liposomes, solid lipid nanoparticles, nanostructured lipid carriers, polymeric nanoparticles, chitosan-based systems, and inorganic nanoplatforms, have demonstrated significant improvements in the solubility, stability, cellular uptake, and oral bioavailability of berberine. However, knowledge gaps remain regarding optimal dosage determination, safety assessment in combination therapy, and establishing efficacy in large-scale clinical trials. Incorporating berberine into combination therapy strategies may improve treatment outcomes, overcome drug resistance, and potentially reduce the toxic burden associated with chemotherapy. Therefore, this review provides a comprehensive analytical framework for berberine’s potential as an adjuvant, elucidates its mechanistic synergistic interactions with standard therapies, explores pharmaceutical strategies to overcome bioavailability limitations, and suggests future research avenues to further its clinical development.

## 1. Introduction

Cancer remains a leading cause of death worldwide, with an estimated 20 million new cases and nearly 9.7 million deaths in 2022 [1]. Despite significant advances in chemotherapy, the effectiveness of treatment remains limited by the emergence of multidrug resistance (MDR), particularly mediated by ATP-binding cassette transporters, and by chemotherapy-associated toxicity [2,3]. Resistance to chemotherapy is a major cause of treatment failure in metastatic cancers, significantly limiting therapeutic effectiveness [4]. Furthermore, conventional chemotherapy is associated with serious side effects, including cardiotoxicity, renal and hepatic toxicity, as well as gastrointestinal disturbances, negatively impacting patients’ quality of life and their ability to continue treatment [5,6].

In this context, growing scientific interest has focused on natural compounds as adjuvant agents capable of enhancing chemotherapy efficacy while mitigating drug resistance and adverse effects, while also exhibiting anti-inflammatory properties that support their use in complementary therapy [7,8]. For centuries, berberine (BBR), a quaternary isoquinoline alkaloid (C_20_H_18_NO_4_) with a molecular weight of 336.36 g/mol, has been used in traditional medicine [9]. Recent extensive preclinical studies have demonstrated that berberine exerts anticancer effects through multiple mechanisms, including induction of apoptosis, cell cycle arrest, modulation of oxidative stress, and inhibition of key molecular signaling pathways [10,11]. Recent studies have demonstrated synergistic effects between berberine and conventional chemotherapeutic agents, enhancing antitumor efficacy and, in combination strategies, potentially reducing chemotherapy-associated toxicity [12,13]. For example, the combination of berberine and doxorubicin has been shown to inhibit breast cancer cell growth more effectively than doxorubicin alone [14]. Berberine has been shown to reverse multidrug resistance in cancer cells, including lung cancer, by modulating MDR mechanisms (MDR-1/P-gp, STAT-3) and to enhance sensitivity to cisplatin in resistant ovarian cancer models, thereby restoring therapeutic responsiveness [15]. However, the clinical application of berberine remains limited due to its extremely low oral bioavailability (below 1%), poor intestinal permeability, self-aggregation in the gastrointestinal tract, and extensive intestinal and hepatic first-pass metabolism [16,17]. Recent advances in nanotechnology and pharmaceutical formulation science have helped overcome some of these limitations by developing improved berberine delivery systems, such as nanoparticles, liposomes, solid lipid nanoparticles, and polymeric systems [18,19]. These nanoformulations demonstrated a significant improvement in the pharmacokinetic properties of berberine, with approximately 3.97-fold higher Cmax and 3.88-fold greater AUC compared to unprocessed berberine [20].

This review aims to provide a comprehensive analysis of current knowledge regarding berberine as an adjuvant in cancer chemotherapy. It examines its chemotherapeutic properties and the challenges associated with its bioavailability, elucidates its synergistic mechanisms with conventional chemotherapeutic agents, discusses its role in overcoming multidrug resistance, reviews pharmaceutical formulation strategies to enhance its pharmacodynamic properties, assesses available clinical evidence and ongoing trials, and identifies prospects for its future development.

## 2. Chemical Structure, Sources, and Extraction of Berberine

### 2.1. Chemical Structure and Classification

**Figure 1:**
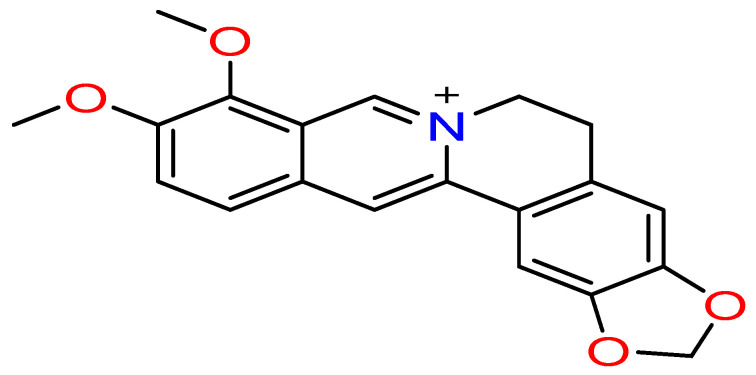
Chemical structure of berberine.

Berberine is a quaternary benzylisoquinoline alkaloid (Figure 1) belonging to the protoberberine structural class and is mainly isolated from the roots, rhizomes, stem, and bark of Berberis vulgaris and other related plant species [21]. Its molecular structure contains a methylenedioxy group at C-2 and C-3 and two methoxy groups at C-9 and C-10, which are critical for its biological activity [22,23]. Berberine bears a quaternary ammonium cation that forms an iminium system (C=N^+^) at the 7-position of the isoquinoline chromophore, conferring a permanent positive charge on the molecule. This structural feature has a dual pharmacological consequence: the positively charged nitrogen at the 7-position plays a leading role in berberine’s ability to form strong complexes with nucleic acids, while the resulting hydrophilicity and lipophobicity hinder crossing of the intestinal plasma membrane, thereby limiting its oral absorption and bioavailability [24,25]. Berberine exhibits photosensitive properties and acts as a natural photosensitizer in photodynamic therapy, generating ROS upon light activation [26]. These structural features combine to determine berberine’s pharmacokinetic properties and its mode of biological action.

### 2.2. Natural Sources and Distribution

Berberine has been detected, isolated, and quantified from various plant families and genera including Annonaceae (Annickia, Coelocline, Rollinia, and Xylopia), Berberidaceae (Berberis, Caulophyllum, Jeffersonia, Mahonia, Nandina, and Sinopodophyllum), Menispermaceae (Tinospora), Papaveraceae (Argemone, Bocconia, Chelidonium, Corydalis, Eschscholzia, Glaucium, Hunnemannia, Macleaya, Papaver, and Sanguinaria), Ranunculaceae (Coptis, Hydrastis, and Xanthorhiza), and Rutaceae (Evodia, Phellodendron, and Zanthoxyllum). The genus Berberis is well-known as the most widely distributed natural source of berberine. The bark of B. vulgaris contains more than 8% of alkaloids, berberine being the major alkaloid (about 5%) [27].

Its content varies significantly with environmental factors such as altitude, season, soil conditions, and plant size, ranging approximately from 0.66% to 7.73% of dry weight in Berberis asiatica [28]. This variation necessitates the adoption of precise standardization procedures in the preparation of pharmaceuticals to ensure the stability of alkaloid content and the reliability of therapeutic efficacy.

### 2.3. Extraction and Purification Techniques

Maceration, percolation, Soxhlet extraction and cold or hot continuous extraction using solvents such as methanol, ethanol and chloroform have been widely applied for berberine isolation as traditional extraction techniques. However, these classical methods have a major drawback, as berberine’s sensitivity to light and heat poses a significant challenge for its extraction, since exposure to high temperature and light could lead to berberine degradation, thus influencing its matrix recovery [27]. To overcome these challenges and improve extraction efficiency, modern methods have been developed.

Supercritical fluid extraction (SFE) utilising carbon dioxide is a sophisticated and selective method that functions at comparatively low temperatures, therefore reducing thermal degradation of bioactive chemicals [29]. Ultra-high-pressure extraction (UPE), typically operating between 100 and 600 MPa, significantly reduces extraction time and is advantageous for thermolabile compounds due to its relatively mild processing temperatures [30]. Microwave-assisted extraction (MAE) significantly shortens extraction time compared with Soxhlet extraction (3 h) and impregnation (7 days), reducing the process to only a few minutes under optimized conditions [31].

Among the more recent developments is Enzyme-assisted extraction (EAE), which employs enzymes such as cellulase and pectinase to degrade plant cell wall components, facilitating the release of target bioactive compounds and enhancing extraction efficiency [32]. In contrast, Ultrasonic-assisted solvent extraction (USE) improves berberine recovery compared with conventional methods such as Soxhlet extraction, significantly shortening extraction time and enhancing yield [31]. Following extraction, berberine samples may undergo purification steps such as liquid–liquid extraction or precipitation, and chromatographic techniques including HPLC and LC-MS/MS are commonly employed for quantitative determination and purity assessment [33,34].

## 3. Pharmacokinetics and Bioavailability of Berberine

### 3.1. Oral Bioavailability and the Basis of Low Systemic Exposure

Berberine is characterized by extremely poor oral bioavailability, generally reported to be below 1%, which represents a major limitation for its broader clinical translation. This low systemic exposure arises from multiple overlapping barriers, including poor membrane permeability, P-glycoprotein (P-gp)-mediated efflux, extensive intestinal and hepatic first-pass metabolism, and marked tissue distribution after absorption, all of which collectively restrict oral absorption and plasma exposure. A physicochemical basis for poor absorption is also suggested by berberine’s quaternary ammonium structure. As a quaternary isoquinoline alkaloid with cationic character, berberine exhibits limited permeability across intestinal membranes, contributing to its low absorption [35,36,37,38]. This limitation is supported by experimental data from Caco-2 models, where the apparent permeability of standard berberine was reported as 4.93 × 10^−6^ cm/s, while an absorption-enhanced formulation increased permeability to 7.18 × 10^−5^ cm/s, indicating that permeability plays a key role in governing berberine absorption [38].

### 3.2. Efflux-Mediated Limitation of Intestinal Absorption

Active intestinal efflux represents an important contributor to berberine’s low absorption. Berberine has been identified as a substrate of P-glycoprotein (P-gp), and P-gp-mediated transport has been reported to play a key role in limiting its oral bioavailability. This is supported by both in vitro and in vivo evidence. A comparative study of protoberberine alkaloids demonstrated that berberine is a P-gp substrate, and in vivo comparison between wild-type and P-gp-knockout mice showed significantly increased systemic exposure in the absence of P-gp, confirming its impact on drug disposition. In Caco-2 monolayers, berberine exhibited net efflux behavior, consistent with limited absorptive transport across the intestinal epithelium [39].

The role of transporter-mediated efflux is further supported by intervention studies aimed at improving berberine exposure. Previous studies have reported that the use of P-gp inhibitors, including TPGS, tetrandrine, silymarin, and gelatin, can enhance berberine absorption and systemic exposure. For example, incorporation of 2.5% TPGS increased berberine Cmax and AUC in rats by 1.9-fold and 2.9-fold, respectively. Similarly, tetrandrine reduced efflux in Caco-2 cells and increased berberine uptake and systemic exposure. These findings are consistent with the contribution of P-gp-mediated efflux as an important factor limiting berberine absorption [36].

### 3.3. Intestinal First-Pass Metabolism as a Dominant Barrier

Beyond poor permeability and efflux, berberine is also subject to extensive presystemic elimination, particularly in the small intestine. This has been directly demonstrated in a rat route-comparison study employing intragastric, intraduodenal, intraportal, and intravenous administration. It was reported that an absolute oral bioavailability of only 0.36% and showed that the dose-normalized AUC following intraduodenal administration was approximately 0.5% of that observed after intraportal dosing. These findings indicate that a substantial fraction of berberine is eliminated within the small intestine before reaching the systemic circulation, whereas gastric first-pass elimination is negligible. The findings indicate that intestinal first-pass elimination represents a major barrier to the oral bioavailability of berberine [40].

This pattern aligns with pharmacokinetic observations indicating that the low systemic exposure of berberine is largely attributed to extensive intestinal first-pass elimination. Approximately half of the orally administered dose of berberine remains unabsorbed and passes intact through the gastrointestinal tract, while the remaining fraction is eliminated within the small intestine before reaching the portal circulation, resulting in extremely low bioavailability. In addition, experimental evidence suggests that approximately half of the administered dose passes through the gastrointestinal tract unchanged, while the remaining fraction is eliminated within the small intestine, further highlighting the intestine as a major barrier to systemic exposure. These observations support the role of intestinal and hepatic metabolism as key determinants governing berberine pharmacokinetics [41].

### 3.4. Phase I and Phase II Metabolism of Berberine

Berberine undergoes extensive biotransformation following oral administration through a series of well-characterized Phase I and Phase II metabolic reactions. Phase I metabolism primarily involves demethylation, demethylenation, hydroxylation, and reduction processes, leading to the formation of key metabolites such as berberrubine, demethyleneberberine, jatrorrhizine, and related derivatives. These transformations are closely linked to the chemical structure of berberine, particularly the methoxy groups and the dioxymethylene ring, which represent major metabolic sites. Notably, demethylation is considered one of the predominant metabolic pathways, generating metabolites with altered physicochemical properties, including increased lipophilicity, which may influence their biological behavior [35].

Following Phase I transformation, berberine metabolites undergo rapid Phase II conjugation reactions, predominantly glucuronidation and, to a lesser extent, sulfation. These conjugation processes significantly increase the polarity of metabolites, facilitating their systemic circulation and eventual elimination. Comprehensive metabolic profiling studies have identified a wide range of berberine-derived metabolites in biological matrices, including plasma, bile, urine, and feces, with reports describing up to 16 metabolites, comprising both Phase I and Phase II products [42].

Importantly, pharmacokinetic analyses indicate that Phase II metabolites constitute the major circulating forms after oral administration, with substantially higher systemic exposure compared to Phase I metabolites. This observation suggests that conjugated metabolites, rather than the parent compound, represent the predominant bioavailable forms in vivo and may contribute significantly to the overall pharmacological effects of berberine [43].

### 3.5. Plasma Exposure, Tissue Distribution, and the “Low Plasma–High Tissue” Pattern

Although berberine exhibits very low plasma levels after oral administration, its tissue distribution is substantially greater than its plasma profile suggests. In rat tissue distribution studies, berberine was rapidly distributed to the liver, kidneys, muscle, lungs, brain, heart, pancreas, and fat, with concentrations in most tissues exceeding plasma levels several hours after dosing. Notably, the AUC in the liver was approximately 10-fold higher than plasma for berberine and up to 30-fold higher for its metabolites [44].

In addition, pharmacokinetic analyses indicate a clear divergence between plasma and tissue kinetics, where berberine is rapidly cleared from systemic circulation but persists for longer durations within tissues. Notably, berberine is capable of crossing the blood–brain barrier and accumulating in brain regions such as the hippocampus, further supporting its favoring tissue distribution over sustained systemic circulation [45].

Consistently, a first-pass pharmacokinetic study demonstrated a marked hepatic predominance, reporting an approximately 70-fold increase in the liver-to-plasma AUC ratio. These findings indicate that low plasma concentrations do not necessarily reflect limited tissue exposure, and that extensive tissue distribution—particularly in the liver—may contribute to the apparent discrepancy between low circulating levels and observed in vivo pharmacological activity. Human data further support the limited systemic exposure of berberine following oral dosing. Previous studies have reported a plasma Cmax of approximately 0.4 ng/mL after a 400 mg oral dose. In a recent pilot study, a standard berberine formulation produced a mean Cmax of 1.67 ± 0.41 ng/mL and an AUC_0–24_ of 13.4 ± 1.97 ng·h/mL, whereas an enhanced LipoMicel formulation increased these parameters by approximately six-fold, reaching a Cmax of 15.8 ± 2.6 ng/mL and an AUC_0–24_ of 78.2 ± 14.4 ng·h/mL. Together, these findings demonstrate that conventional berberine formulations result in low systemic exposure in humans, while formulation strategies can substantially improve pharmacokinetic performance [38,40,44].

### 3.6. Excretion Profile

Experimental data indicate that excretion plays a critical role in the markedly reduced bioavailability of berberine following oral administration. In a comprehensive pharmacokinetic study in rats, 16 metabolites—including phase I (demethylated derivatives) and phase II (glucuronide and sulfate conjugates) were identified, reflecting extensive metabolism prior to elimination. The total recovery of berberine and its metabolites reached 22.83% within 48 h, with a clear predominance of fecal excretion (22.74% of the administered dose), of which approximately 84% consisted of unchanged berberine prototype, whereas urinary (≈0.0939%) and biliary excretion were minimal [42]. Thalifendine and berberrubine were identified as the major urinary metabolites, while most of the parent compound was recovered unchanged in feces, indicating limited absorption and a dominant role for intestinal elimination. In addition, berberine undergoes hepatic metabolism, primarily via demethylation and glucuronidation, followed by biliary secretion and partial reabsorption through enterohepatic circulation, with gut microbiota contributing to metabolite recycling. Only trace amounts of unchanged berberine are eliminated in urine, whereas conjugated metabolites (sulfate and glucuronide conjugates) represent the predominant forms excreted in urine, whereas fecal excretion is dominated by the unchanged parent compound (~84%) [45]. Overall, extensive fecal elimination driven by poor absorption, intensive hepatic phase I/II metabolism, and partial enterohepatic cycling with net fecal loss at each pass represents a major pathway contributing to the markedly low systemic exposure of berberine after oral administration.

## 4. Molecular Mechanisms Underlying the Anticancer Activity of Berberine

Berberine exerts anticancer activity through a pleiotropic pharmacological profile that does not depend on a single dominant molecular target. Its antitumor effects repeatedly converge on three interrelated processes: induction of apoptosis, interruption of cell-cycle progression, and modulation of major survival and stress-responsive signaling pathways. Importantly, these responses are not identical in all tumor models. Instead, berberine acts in a context-dependent manner, with the dominant outcome varying according to cancer type, intracellular accumulation, dose, treatment duration, and molecular background, especially p53 status. This point is supported by comparative studies showing that berberine can provoke G0/G1, S, or G2/M arrest, activate caspase-dependent apoptosis to different extents, and alter distinct transcriptional programs in different cell lines. Thus, the most accurate interpretation is that berberine functions as a multi-target regulator of tumor cell fate rather than as a single-pathway inhibitor [46,47,48].

### 4.1. Apoptosis Induction

The most consistently reported pharmacological action of berberine is induction of apoptotic cell death, particularly through the mitochondrial pathway, although death-receptor-associated signaling is also involved in some models [49,50]. In HepG2 hepatoma cells, berberine caused internucleosomal DNA fragmentation, loss of mitochondrial membrane potential, cytochrome c release, activation of caspase-8 and caspase-3, PARP cleavage, activation of Fas, cleavage of Bid, and downregulation of the anti-apoptotic protein Bcl-XL; moreover, pharmacologic inhibition of caspases significantly attenuated berberine-induced apoptosis, indicating that caspase activation plays a critical mechanistic role in this process [49]. In HSC-3 oral cancer cells, berberine increased ROS generation and intracellular Ca^2+^ levels, disrupted mitochondrial membrane potential, and promoted cytochrome c release together with activation of caspase-3 [51]. In parallel, berberine enhanced Fas-associated signaling and caspase-8 activation, alongside modulation of mitochondrial apoptotic proteins, supporting the concurrent involvement of intrinsic and extrinsic apoptotic pathways. In SW620 colon carcinoma cells, berberine-induced apoptosis was associated with reactive oxygen species (ROS) generation, mitochondrial cytochrome c release, activation of caspase-8 and caspase-3, PARP cleavage, and downregulation of anti-apoptotic proteins including c-IAP1, Bcl-2, and Bcl-XL [50]. In addition, evidence from multi–cancer cell line studies indicates that berberine consistently increases BAX expression while suppressing BCL-2, thereby shifting the BAX/BCL-2 balance toward apoptosis and suggesting that modulation of the Bcl-2 family represents a common mechanistic endpoint across different tumor types [52]. Taken together, these studies indicate that berberine does not merely trigger nonspecific cytotoxicity; rather, it shifts the intracellular balance from survival to apoptosis by destabilizing mitochondria, activating caspase cascades, and suppressing anti-apoptotic buffering mechanisms.

### 4.2. Cell-Cycle Arrest and Proliferation Inhibition

In parallel with apoptosis, berberine suppresses tumor cell proliferation by arresting cells at critical checkpoints, but the specific phase of arrest is clearly model-dependent. These effects have been linked to the downregulation of key cell-cycle regulators, including cyclins (Cyclin D1, Cyclin E, and Cyclin B1) and cyclin-dependent kinases, as well as inhibition of retinoblastoma (Rb) phosphorylation, thereby preventing E2F release and blocking the G1-to-S phase transition [53]. In human osteosarcoma cells, berberine induced both G1 and G2/M arrest, with G1 arrest being p53-dependent and accompanied by upregulation of p21, p27, and pro-apoptotic genes, whereas G2/M arrest occurred even when p53 function was impaired. That same study demonstrated accumulation of DNA double-strand breaks, reflected by increased γ-H2AX, suggesting that genotoxic stress is an upstream event capable of activating p53-linked checkpoint responses [48]. Berberine arrested MIA PaCa-2 pancreatic cancer cells mainly in G1, whereas U343 glioblastoma cells were arrested in G2, again emphasizing cell-specific behavior [47]. In melanoma A375 cells, lower berberine concentrations induced S- and G2/M-phase accumulation, whereas higher concentrations shifted cells predominantly into G2/M arrest; this was accompanied by increased miR-582-5p and miR-188-5p and decreased CDK1, CDK2, cyclin D1, and cyclin A at both mRNA and protein levels, suggesting that berberine can regulate cell-cycle progression partly through miRNA-mediated post-transcriptional control [54]. In multiple cancer models, including breast, cervical, and colon cancer cells, berberine has been reported to induce cell cycle arrest—often at the G2/M phase—alongside apoptosis and modulation of BAX/BCL-2 expression [55]. Collectively, these findings show that berberine perturbs the cyclin-CDK machinery at multiple nodes, commonly involving p53, p21, p27, cyclins, and CDKs, but without enforcing a universal checkpoint signature in every tumor type. This variability is important to acknowledge, as it helps explain why berberine may exhibit predominantly cytostatic effects in some cancer types while demonstrating more pronounced pro-apoptotic activity in others.

### 4.3. Modulation of Signaling Pathways

The upstream pharmacology of berberine is best understood as modulation of multiple interconnected signaling networks that regulate survival, stress responses, inflammation, metabolism, and apoptosis. Among these, the PI3K/Akt/mTOR axis appears particularly important. A recent study in oral squamous cell carcinoma demonstrated that berberine reduced RAGE expression and decreased the phosphorylation levels of PI3K, Akt, and mTOR, thereby inhibiting cell proliferation and migration while promoting apoptosis, supporting suppression of a key prosurvival signaling pathway in OSCC. In addition, evidence from systems-level analyses and anticancer studies indicates that berberine is associated with reduced Akt phosphorylation, suppression of mTOR signaling, and enhancement of downstream pro-apoptotic responses [56,57]. AMPK is another major node. Berberine has been shown to inhibit colon tumorigenesis through regulation of AMPK signaling pathways. In addition, accumulating evidence indicates that AMPK activation links berberine to reduced anabolic growth, suppression of mTOR signaling, and, in certain contexts, the concurrent induction of apoptosis and autophagy [57,58].

**Figure 2:**
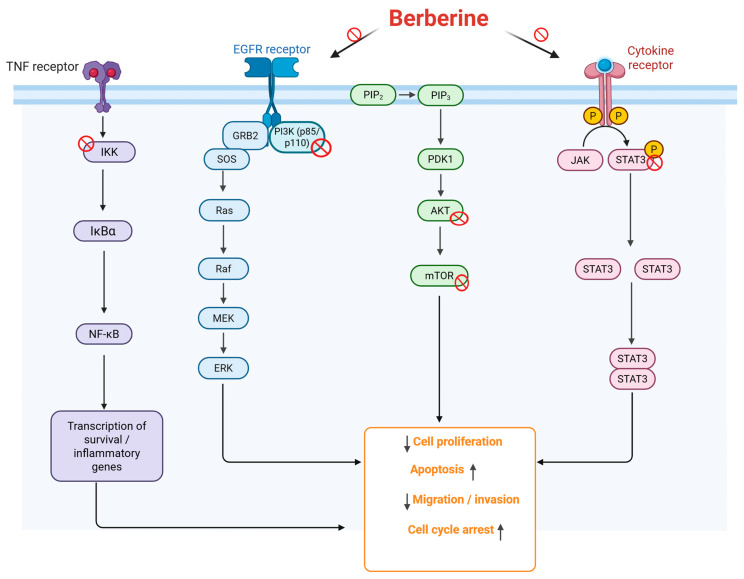
Signaling pathways modulated by berberine. Created in BioRender. Alloush, T. (2026) https://BioRender.com/6r1ae8e, (accessed on 4 April 2026).

Importantly, AMPK functions as a central energy sensor integrating metabolic stress with downstream signaling cascades, and its activation by berberine has been associated with modulation of oxidative stress, inflammation, and lipid and glucose metabolism, thereby reinforcing its role as a master regulator of cellular homeostasis [59]. Beyond these canonical survival and metabolic pathways, berberine exerts significant regulatory effects on inflammatory signaling networks. In particular, the NF-κB pathway represents a major molecular target. Mechanistically, berberine suppresses NF-κB activation through multiple coordinated steps, including inhibition of IκBα phosphorylation and degradation, suppression of IKK activity, and interference with p65/p50 nuclear translocation, ultimately leading to reduced transcription of pro-inflammatory cytokines such as TNF-α, IL-1β, and IL-6. Notably, this inhibitory effect is closely linked to upstream modulation of PI3K/Akt signaling, highlighting the functional crosstalk between inflammatory and prosurvival pathways. In parallel, berberine modulates the mitogen-activated protein kinase (MAPK) signaling cascade, including ERK, JNK, and p38 pathways, which are critical regulators of cellular stress responses and proliferation. Evidence indicates that berberine inhibits MAPK activation by suppressing upstream kinases such as MEK and ASK1, thereby attenuating inflammatory mediator production and limiting proliferative responses [60,61]. Figure 2 illustrates the major signaling pathways modulated by berberine, including NF-κB, EGFR/Ras/Raf/MEK/ERK, PI3K/AKT/mTOR, and JAK/STAT3, which collectively contribute to reduced cell proliferation and migration/invasion, as well as enhanced apoptosis and cell cycle arrest.

### 4.4. Autophagy and Autophagic Cell Death

The autophagy-inducing activity of berberine extends across multiple cancer types through distinct yet interconnected signaling pathways. In glioblastoma cells, autophagy is activated by targeting the AMPK/mTOR/ULK1 axis. In breast cancer cells, autophagic death is promoted through JNK-mediated phosphorylation, which drives the dissociation of the Bcl-2/Beclin-1 inhibitory complex, thereby liberating Beclin-1 to initiate autophagosome formation. An identical mechanism of Beclin-1 release from the Bcl-2/Beclin-1 complex occurs in hepatoma cells. Additionally, the interaction between GRP78 and VPS34, a key lipid kinase in autophagy initiation, is enhanced by berberine, further amplifying the autophagic signal. SREBP-1 expression is also indirectly suppressed via AMPK activation, which subsequently engages the ULK1/mTOR1 signaling axis to further promote autophagy. Notably, the capacity to regulate autophagy through AMPK has been identified as a mechanism capable of reversing drug resistance in cancer cells, underscoring its potential clinical relevance [53]. Direct experimental evidence corroborates these mechanistic frameworks, demonstrating that the induction of autophagy by berberine exhibits a tumor-selective response. Treatment with berberine induces autophagy specifically in U343 glioblastoma and MIA PaCa-2 pancreatic carcinoma cell lines, while no autophagic activity is observed in normal human dermal fibroblasts (HDF). This tumor-selective induction has been confirmed via gene-expression analysis and fluorescence-based assays, revealing the upregulation of the autophagosome-associated LC3 isoform alongside elevated Beclin1 (BECN1) expression. Furthermore, Death-Associated Protein 1 (DAP1), a negative regulator of autophagy, is upregulated in these tumor cells following treatment, suggesting the activation of a feedback regulatory mechanism within the autophagic process. The AMPK/mTOR/ULK1 pathway is similarly implicated as the primary signaling axis mediating this targeted autophagy induction in glioblastoma cells [47]. In doxorubicin-resistant MCF-7/ADR breast cancer cells, for instance, berberine inhibits autophagosome formation by blocking LC3-II accumulation and promoting the cellular sequestration of p62, operating through the PTEN/Akt/mTOR signaling axis, an effect that effectively restores chemosensitivity to doxorubicin [55].

This bidirectional regulatory capacity is not a mechanistic inconsistency but rather reflects berberine’s ability to sense and counteract the dominant autophagic state of the tumor: inducing autophagy where it is deficient to trigger cell death and suppressing it where it is overactivated to dismantle a key resistance mechanism. In both cases, the net oncological outcome remains anti-tumorigenic, achieved through mechanistically opposite but contextually appropriate interventions.

## 5. Modulation of Multidrug Resistance: Role of ABC Transporters

Berberine exhibits a clear interaction with ATP-binding cassette (ABC) transporters, particularly those associated with multidrug resistance. Quantitative evidence from a large-scale analysis of the NCI-60 tumor cell lines demonstrated that berberine IC_50_ values significantly correlate with classical multidrug resistance-associated drugs such as daunorubicin, vinblastine, and paclitaxel, as well as with the expression of several ABC transporter genes. Functional validation in an ABCB1 (P-glycoprotein)-overexpressing leukemia model (CEM/VCR1000) revealed a pronounced resistance phenotype, confirming that ABCB1 contributes directly to berberine resistance [62].

Mechanistically, experimental evidence indicates that berberine transport is ATP-dependent and sensitive to P-glycoprotein inhibitors, as uptake into Coptis japonica cells was strongly and dose-dependently inhibited by MDR/P-gp inhibitors. In parallel, ATP-dependent photolabeling experiments identified ABC transporter proteins that interact with berberine, suggesting the involvement of MDR-type ABC transporters in its cellular transport [63]. Experimental and functional analyses indicate that ABC transporters recognize berberine as a substrate and contribute to its cellular uptake and, thereby, indirectly to its vacuolar accumulation, while transport directionality and substrate specificity may vary depending on the biological system [64]. Functional evidence demonstrates that berberine is actively effluxed by both P-glycoprotein (ABCB1) and MRP1. In MDR1- or MRP1-overexpressing cells, intracellular berberine accumulation is markedly reduced compared with control cells, and this effect is reversed by classical transporter inhibitors such as verapamil and cyclosporine A. Moreover, berberine efflux is ATP-dependent, confirming that it behaves as a substrate of ABC transporters and linking its reduced intracellular concentration to multidrug resistance mechanisms [65].

## 6. Berberine–Chemotherapy Combination Efficacy in Specific Cancer Types

**Figure 3:**
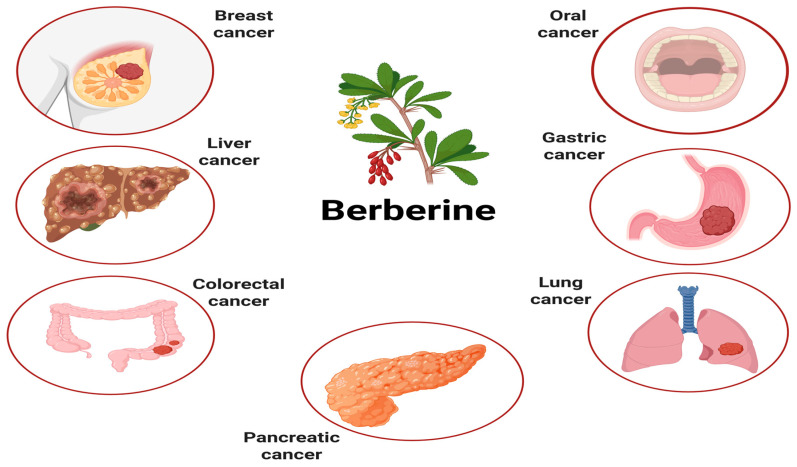
Major cancer types in which berberine has demonstrated therapeutic potential. Created in BioRender. Alloush, T. (2026) https://BioRender.com/wy03ht2, (accessed on 4 April 2026).

Figure 3 summarizes the major cancer types targeted by berberine and the associated molecular mechanisms involved.

### 6.1. Breast Cancer

Triple-negative breast cancer (TNBC), characterized by the absence of estrogen (ER) and progesterone (PR) receptors and the lack of HER2 receptor overexpression, is one of the most aggressive clinical types, with a clear limitation in available treatment options [66,67]. Studies have shown that berberine exerts significant pro-apoptotic and anti-proliferative effects in breast cancer through multiple molecular targets and signaling pathways [68]. In Berberine exhibited strong anti-proliferative activity in TNBC cell lines such as HCC70 and MDA-MB-468, with IC_50_ values of 0.19 and 0.48 µM, respectively, whereas MDA-MB-231 cells were more resistant (IC_50_ = 16.7 µM) [69].

- Berberine and Doxorubicin Combination

Combination strategies involving berberine and doxorubicin have been increasingly investigated to enhance therapeutic efficacy while reducing toxicity in breast cancer models. In a study using MCF-7 cells, dual-loaded PCL nanofibers containing both agents demonstrated significantly enhanced cytotoxicity compared to single-drug systems, accompanied by downregulation of anti-apoptotic BCL-2 and upregulation of caspase-3 indicating apoptosis induction; importantly, the combination approach has the potential to reduce the systemic toxicity typically associated with doxorubicin while maintaining therapeutic efficacy [70].

Similarly, pH-sensitive chondroitin sulfate-based nanomicelles co-delivering berberine and doxorubicin showed superior antitumor activity in both in vitro co-culture models (MCF-7 + fibroblasts) and in vivo in 4T1 tumor-bearing mice, where the system enhanced tumor accumulation, promoted apoptosis, inhibited angiogenesis and metastasis, and reduced toxicity and side effects, as indicated by improved biocompatibility and reduced organ damage [71].

Furthermore, a biomimetic co-assembled nanodrug combining berberine with doxorubicin demonstrated that, in 4T1 cells and orthotopic tumor models, doxorubicin alone could paradoxically enhance metastasis via HMGB1–TLR4 signaling, whereas the addition of berberine effectively suppressed this effect, leading to reduced tumor growth and pulmonary metastasis with fewer side effects in vivo [72]. Notably, another study indicated that berberine does not significantly interfere with the cytotoxic efficacy of doxorubicin in MCF-7 cells while providing cardioprotective effects in vitro and in vivo (rat models), supporting the concurrent preservation of anticancer activity alongside attenuation of doxorubicin-induced toxicity [73].

- Berberine and Tamoxifen Combination

Berberine has been investigated as a sensitizing agent to enhance the therapeutic efficacy of tamoxifen in breast cancer, particularly in resistant phenotypes. In estrogen receptor-positive models such as MCF-7 and tamoxifen-resistant MCF-7/TAM, combination treatment with berberine and tamoxifen significantly reduced cell viability and proliferation compared to tamoxifen alone, accompanied by enhanced apoptosis and G1 phase cell cycle arrest through upregulation of p21 and modulation of the Bcl-2/Bax axis [74]. Mechanistically, berberine was shown to sensitize resistant cells (including MCF7-TAMR and BT-474) by downregulating ER-α36 expression and disrupting the ER-α36/EGFR/HER2 signaling loop, thereby restoring responsiveness to tamoxifen therapy. Cytotoxicity analysis using MTT assays demonstrated that berberine alone exhibited inhibitory effects with IC_50_ values of 20.77 µM in MCF7-TAMR cells and 24.28 µM in BT-474 cells, while the combination treatment produced a synergistic suppression of cell growth over a 7-day period [75]. Furthermore, berberine exhibited relatively selective cytotoxicity toward cancer cells, with limited effects on normal breast epithelial cells (MCF-12F) at lower concentrations [76]. Overall, these findings indicate that berberine enhances tamoxifen efficacy by overcoming resistance mechanisms, promoting apoptosis, and improving therapeutic selectivity in breast cancer models.

### 6.2. Gastric Cancer

Gastric cancer is one of the deadliest cancers globally, due to its high mortality rates, limited treatment options, and the frequent development of chemotherapy resistance [77]. Studies have shown that berberine exhibits anticancer activity through multiple mechanisms, including inhibition of matrix metalloproteinases (MMPs) and enhancement of reactive oxygen species (ROS) generation, which contributes to impairing the ability of cancer cells to invade and survive [78,79].

- Berberine and Cisplatin Combination

Berberine has been shown to enhance cisplatin sensitivity in gastric cancer, particularly in resistant models such as SGC-7901/DDP and BGC-823/DDP. Treatment with berberine (10 μM) partially reversed cisplatin resistance by promoting caspase-dependent apoptosis and upregulating miR-203, which targets the anti-apoptotic protein Bcl-w. Cytotoxicity was assessed using MTT-based dose–response analysis, where IC_50_ values were derived from survival curves and indicated reduced resistance following berberine exposure [80].

Berberine significantly sensitized cisplatin-resistant gastric cancer cells (BGC-823/DDP and SGC-7901/DDP) to cisplatin, with 10 and 30 μM concentrations markedly reducing IC_50_ values. This effect was associated with enhanced apoptosis in the combination group, as evidenced by increased activation of caspase-3 and caspase-9, along with downregulation of drug resistance-related proteins MDR1 and MRP1. Mechanistically, these effects were linked to suppression of the PI3K/AKT/mTOR signaling pathway. In vivo, co-administration of berberine (10 mg/kg/day) with cisplatin (3 mg/kg/day) in SGC-7901/DDP xenograft-bearing mice suppressed tumor growth by approximately 50% and significantly increased apoptotic activity compared with cisplatin alone [81].

- Berberine and 5-Fluorouracil Combination

Berberine demonstrated dose-dependent cytotoxic effects in human gastric adenocarcinoma AGS cells, with an IC_50_ of approximately 29.2 µM. In combination with 5-fluorouracil (5-FU), berberine exhibited synergistic inhibition of cell viability compared to either agent alone. Mechanistically, this combination markedly suppressed STAT3 activation and downregulated survivin expression, key mediators of chemoresistance in gastric cancer. This effect was associated with enhanced apoptotic cell death, as evidenced by increased Annexin V staining and activation of caspases [82].

### 6.3. Colorectal Cancer

Berberine has demonstrated multifaceted anticancer activity in colorectal cancer (CRC) through both in vitro and in vivo evidence, primarily by targeting proliferation, apoptosis, inflammation, and tumor microenvironment regulation. Mechanistically, berberine inhibits CRC cell growth by inducing cell cycle arrest and apoptosis via pathways such as AMPK/mTOR, NF-κB suppression, and caspase activation, while also reducing anti-apoptotic proteins (e.g., Bcl-2) and promoting ROS-mediated mitochondrial dysfunction in colon cancer cell lines (e.g., IMCE and other CRC models). Importantly, these cytotoxic effects appear relatively selective for tumor cells, as normal colon epithelial cells show lower sensitivity. In vivo studies further confirm that berberine activates AMPK signaling, suppresses tumor growth, and modulates inflammatory mediators and oncogenic pathways, including IL-6/STAT3 and NF-κB. Additionally, berberine regulates gut microbiota composition and reduces pro-inflammatory cytokines (e.g., TNF-α, IL-1β), thereby limiting CRC progression and improving intestinal barrier function in animal models [83,84].

- Berberine and Cisplatin Combination

In colorectal cancer models, the combination of berberine with cisplatin has been investigated in human colon cancer cell lines such as HCT116 and RKO, particularly within the context of a rationally designed Pt(IV) prodrug (berplatin) or co-treatment strategy. Mechanistically, berberine enhances cisplatin-mediated cytotoxicity through increased DNA damage and apoptosis, as evidenced by elevated γH2AX levels, p53 activation, Bcl-2 downregulation, and caspase-3 cleavage, along with induction of cell cycle arrest. In vitro, co-treatment with berberine and cisplatin (Ber + cDDP) showed slightly improved antiproliferative effects compared to either agent alone (IC_50_ ≈ 8.0 µM vs. ≈10.0 µM for cisplatin and ≈9.0 µM for berberine in HCT116 cells), indicating a modest enhancement. Notably, the berplatin conjugate exhibited markedly enhanced intracellular platinum accumulation via endocytosis-dependent pathways, leading to significantly greater DNA damage and cytotoxicity. In vivo, berplatin demonstrated superior antitumor efficacy in HCT116 xenograft models and reduced systemic toxicity compared to cisplatin. Furthermore, normal intestinal epithelial cells (HIEC-6) exhibited substantially higher IC_50_ values (>50 µM), supporting the tumor selectivity and improved safety profile of berberine-based platinum strategies [85].

- Berberine and Irinotecan Combination

Berberine has been shown to enhance the chemosensitivity of irinotecan (CPT-11) in colorectal cancer models. In vitro studies using human colorectal cancer HCT116 cells demonstrated that berberine (2.5–10 µM, non-cytotoxic range) significantly increased CPT-11-induced apoptosis, raising the inhibition rate from ~11% with CPT-11 alone (20 µM) to ~49% in combination. Mechanistically, this effect was mediated by suppression of NF-κB activation and subsequent downregulation of anti-apoptotic proteins, including c-IAP1, c-IAP2, survivin, and Bcl-xL [86].

Additional mechanistic support comes from colorectal models (Caco-2 and CT26), where berberine improved epithelial barrier integrity and attenuated SN-38–induced damage by enhancing tight junction protein expression and reducing permeability. In vivo, berberine (50 mg/kg, oral) alleviated irinotecan-induced intestinal mucositis, including mucosal injury, inflammation, and barrier dysfunction, while preserving its antitumor efficacy in CT26 tumor-bearing mice. Mechanistically, these protective effects were associated with inhibition of bacterial β-glucuronidase activity, leading to reduced intestinal SN-38 levels. These findings highlight the potential of berberine to mitigate gastrointestinal toxicity without compromising the anticancer activity of irinotecan [87].

### 6.4. Pancreatic Cancer

Berberine (BBR) has demonstrated notable anticancer activity in pancreatic cancer, primarily supported by in vitro studies on pancreatic ductal adenocarcinoma (PDAC) cell lines, including MIA-PaCa-2 and other human pancreatic adenocarcinoma models. Mechanistically, BBR suppresses cancer cell proliferation through activation of AMPK and inhibition of key oncogenic pathways such as PI3K/AKT/mTOR and RAF/MEK/ERK, alongside modulation of reactive oxygen species (ROS), induction of apoptosis, and interference with gene expression via DNA binding and epigenetic regulation [88,89]. Additionally, exposure of BxPC-3 pancreatic adenocarcinoma cells and non-malignant HPDE-E6E7c7 pancreatic ductal epithelial cells to berberine (10–200 µM) for 24–72 h resulted in a concentration- and time-dependent reduction in cell proliferation and induction of caspase-independent cell death [90].

- Berberine and Gemcitabine Combination

The combination of berberine with gemcitabine exhibits enhanced anticancer efficacy in pancreatic ductal adenocarcinoma (PDAC), with particularly pronounced effects in gemcitabine-resistant (Gem-R) derivatives of MIA PaCa-2 and BxPC-3 cells. Berberine alone showed moderate cytotoxicity, with IC_50_ values of 16.5–19.0 µg/mL in parental cells and 14.6–24.8 µg/mL in Gem-R cells, while gemcitabine exhibited IC_50_ values of approximately 588–618 nM in parental cells. Notably, combination treatment significantly enhanced cytotoxicity, reducing IC_50_ values to 9.2–12.1 µg/mL for berberine and 138–181 nM for gemcitabine in parental cells, and to 8.2–17.1 µg/mL and 123–257 nM, respectively, in resistant cells, with combination index (CI) values < 1 (0.45–0.82), indicating synergism. Functionally, co-treatment enhanced inhibition of cell viability, clonogenicity, migration, and invasion, while inducing G0/G1 cell cycle arrest and apoptosis, with increased apoptotic rates (e.g., ~35.5% vs. ~11–21% with single agents). These effects were associated with suppression of the Rap1/PI3K-Akt signaling pathway, a key regulator of gemcitabine resistance [91].

### 6.5. Lung Cancer

Berberine has demonstrated significant anticancer activity against lung cancer in both in vitro and in vivo models, particularly in non-small cell lung cancer (NSCLC). In vitro, studies using A549 (p53 wild-type) and H1299 (p53-deficient) lung cancer cell lines showed that berberine inhibits cell proliferation in a dose- and time-dependent manner and induces apoptosis through mitochondrial dysfunction, characterized by downregulation of Bcl-2/Bcl-xl, upregulation of Bax/Bak, and activation of caspase-3 [92].

In vivo, berberine administration in tumor xenograft and murine lung cancer models significantly suppressed tumor growth, further confirming its therapeutic potential. Complementary evidence from recent reviews indicates that berberine consistently exerts anti-proliferative, pro-apoptotic, anti-migratory, and cell cycle arrest effects in lung cancer models, including modulation of key pathways such as PI3K/AKT, MAPK, and ROS-mediated signaling, and that its efficacy can be enhanced via nanoparticle delivery systems [93].

However, clinical translation is still limited by poor bioavailability and rapid metabolism, which may affect systemic exposure despite promising preclinical efficacy and relatively low toxicity reported in experimental studies [94].

- Berberine and EGFR-TKIs Combination

The co-administration of berberine with EGFR-TKIs has demonstrated enhanced therapeutic efficacy in lung cancer, particularly in resistant non-small cell lung cancer (NSCLC) models. In EGFR-TKI-resistant NSCLC cell lines (H460 and H1299), the combination of berberine with the first-generation EGFR-TKI icotinib produced a strong synergistic antiproliferative effect, with combination index (CI) values generally <0.7, indicating robust synergy. Although individual IC_50_ values varied depending on the cell line (e.g., icotinib IC_50_ ~ 158–164 µM in resistant cells), the combination significantly enhanced cytotoxicity, suppressed colony formation, and induced both autophagic cell death and apoptosis, accompanied by increased ROS generation and inhibition of EGFR signaling. Importantly, toxicity toward normal bronchial epithelial BEAS-2B cells was minimal, as neither berberine nor the combination significantly affected their viability, indicating good selectivity. These findings were further validated in an in vivo xenograft mouse model, where co-administration of berberine (80 mg/kg) and icotinib (125 mg/kg) markedly inhibited tumor growth compared to either agent alone, without significant body weight loss [95].

Complementary evidence using the third-generation EGFR-TKI osimertinib demonstrated that berberine can sensitize MET-amplified osimertinib-resistant NSCLC cells (e.g., HCC827/AR models), where the combination synergistically decreased cell survival and enhanced apoptosis through Bim upregulation and Mcl-1 downregulation. These effects were further confirmed in xenograft models, where the combination significantly suppressed tumor growth with good tolerability [96].

Additionally, mechanistic studies indicate that berberine can reverse osimertinib resistance in NSCLC by targeting Notch1 signaling and suppressing EMT, thereby restoring sensitivity to osimertinib, with minimal reported toxicity [97].

- Berberine and Cisplatin Combination

In NSCLC H1299 cells, the combination of berberine and cisplatin significantly enhanced cytotoxicity compared to individual treatments. Both agents reduced cell viability in a dose- and time-dependent manner, with IC_50_ values of 25.5 ± 3.21 µM for berberine and 23.76 ± 0.39 µM for cisplatin after 48 h. At a 1:4 molar ratio (berberine/cisplatin), the combination exhibited synergistic effects (CI = 0.70). Mechanistically, co-treatment markedly increased apoptosis (~57% vs. ~28% with cisplatin alone), mitochondrial membrane depolarization, and caspase-3 activation, associated with activation of the p38-MAPK signaling pathway. Notably, the combination demonstrated preferential toxicity toward cancer cells compared to normal bronchial epithelial cells. Importantly, regarding toxicity, berberine alone exhibited minimal cytotoxicity toward normal bronchial epithelial BEAS-2B cells, whereas cisplatin significantly reduced their viability. Notably, the combination maintained selective cytotoxicity toward cancer cells, with a selectivity index (SI) of ~1.8–2.4, indicating preferential tumor targeting and reduced impact on normal cells [98].

### 6.6. Oral Cancer

Berberine exhibits anti-inflammatory activity in oral cancer models through modulation of prostaglandin synthesis pathways. In human oral cancer cell lines (OC2 and KB), berberine (1, 10, and 100 μM) reduced prostaglandin E_2_ (PGE_2_) production in a dose-dependent manner by suppressing COX-2 protein expression, without inhibiting COX-1 or COX-2 enzymatic activity. Mechanistically, this effect was associated with inhibition of AP-1 DNA-binding activity. Importantly, these effects were not accompanied by cytotoxicity within the tested conditions (2–12 h), indicating that the reduction in PGE_2_ and COX-2 expression was not due to nonspecific cytotoxic effects. In vivo, berberine also significantly reduced PGE_2_ production and inflammatory exudate formation in a carrageenan-induced rat model [99].

Complementary evidence from human oral tongue squamous carcinoma SCC-4 cells demonstrated that berberine induces apoptosis and inhibits tumor growth. In a murine xenograft model, intraperitoneal administration of berberine (10 mg/kg) significantly suppressed tumor growth, achieving approximately 52% tumor inhibition compared with controls. Notably, no observable systemic toxicity was reported, as indicated by stable body weight and normal behavior in treated animals. In vitro, higher concentrations of berberine (~30 μM) were associated with substantial cytotoxic effects in SCC-4 cells, including over 50% reduction in cell viability and induction of apoptosis, although no explicit IC_50_ values were reported [100].

- Berberine and 5-Fluorouracil Combination

The combination of berberine (BER) and 5-fluorouracil (5-FU) demonstrated synergistic effects in oral cancer models using AW13516 cells. In vitro cytotoxicity (MTT assay) showed that both agents, individually and in equimolar combinations (1, 2, 5, and 10 μM), reduced cell viability in a dose-dependent manner. Combination index (CI) values <1 at 1–5 μM confirmed synergistic interactions, while an additive effect was observed at 10 μM. Notably, the dual drug-loaded nanoemulsion-loaded mucoadhesive gel significantly enhanced apoptosis, with treatment equivalent to 2 μM 5-FU inducing ~48% apoptosis compared to ~9% with 5-FU alone. In CD44^+^ cancer stem-like cells, the formulation reduced cell viability by approximately 2.5-fold relative to 5-FU alone. In vivo biocompatibility studies in New Zealand rabbits demonstrated no significant mucosal irritation after 7 days of application, with only minimal histological changes observed. This favorable safety profile has been suggested to be associated with the presence of berberine and reduced local exposure to 5-FU [101].

### 6.7. Liver Cancer

Berberine exhibits anticancer activity in hepatocellular carcinoma (HCC), as demonstrated in both in vitro and in vivo models. In human hepatoma cell lines (HepG2 and Bel-7404) and murine H22 cells, berberine reduced cell viability in a dose- and time-dependent manner and induced apoptosis via a caspase-independent mechanism involving apoptosis-inducing factor (AIF) translocation. Mechanistically, these effects were associated with suppression of cytosolic phospholipase A2 (cPLA2) and cyclooxygenase-2 (COX-2), leading to modulation of the arachidonic acid pathway and an increased AA/PGE2 ratio. Reported IC_50_ values after 72 h treatment were approximately 9.21 µM (Bel-7404), 43.2 µM (H22), and 82.8 µM (HepG2), whereas normal hepatic HL-7702 cells exhibited a higher IC_50_ (~122.4 µM), indicating selective cytotoxicity toward cancer cells. In vivo, berberine reduced tumor volume and weight in an H22 transplanted tumor model in mice following oral administration (12.5–50 mg/kg), demonstrating dose-dependent antitumor activity [102].

Mechanistically, berberine induces both apoptotic and autophagic cell death in HepG2 cells through activation of the AMPK pathway and subsequent inhibition of mTORC1 signaling. These effects are concentration-dependent, with higher concentrations (50–100 µM) significantly reducing cell viability and increasing cell death, whereas lower concentrations (~10 µM) exert minimal effects [103]. Berberine exhibits broad antitumor effects in liver cancer through modulation of oxidative stress, inflammation, apoptosis, and metastasis-related pathways, with experimental studies suggesting a favorable safety profile [104].

## 7. Berberine Toxicity

Berberine exhibits dose and route-dependent toxicity. Acute toxicity studies in mice demonstrated LD_50_ values of 9.04 mg/kg (intravenous) and 57.61 mg/kg (intraperitoneal), whereas no definitive LD_50_ could be established after oral administration due to extremely low bioavailability and limited systemic absorption. Toxicity was strongly correlated with blood concentration, with oral doses ≥41.6 g/kg associated with increased mortality and plasma levels around 0.432 μg/mL. Berberine may induce gastrointestinal irritation, ulceration, immunotoxicity, neurotoxicity, cardiotoxicity, and jaundice in a dose-dependent manner, and special caution is advised during pregnancy, in neonates, and in individuals with G6PD deficiency. Additionally, berberine inhibits several CYP enzymes (including CYP2D6 and CYP3A4), suggesting potential pharmacokinetic interactions when co-administered with other drugs [105,106].

Berberine inhibits mitochondrial respiration, leading to reduced ATP levels and an increased NADH/NAD^+^ ratio. These effects are associated with impaired hepatic gluconeogenesis and ammonia detoxification, reflecting a disruption of energy metabolism [107]. Berberine exhibits ocular toxicity in sensitive systems, particularly in human corneal epithelial cells and zebrafish models. In human corneal epithelial cells, berberine and its metabolite berberrubine showed concentration-dependent cytotoxicity (IC_50_ ≈ 301.45 and 231.88 µM, respectively), while in zebrafish, berberine demonstrated significant toxicity (LC_50_ ≈ 164.7 µM). Mechanistically, these effects were associated with mitochondrial dysfunction, oxidative stress, autophagic dysfunction, apoptosis, and inflammation [108].

Conversely, in pathological conditions, berberine may exert protective effects, as demonstrated in models of gentamicin-induced nephrotoxicity, where it attenuates oxidative stress, inflammation, and mitochondrial damage [109].

This apparent duality highlights that berberine’s toxicity is closely linked to its bioenergetic and redox-modulating properties, which may shift from therapeutic to deleterious depending on dose, exposure duration, and tissue context.

## 8. Pharmaceutical Formulations and Bioavailability Enhancement

### 8.1. Conventional Formulations

The purified extract of berberine is subsequently processed into oral pharmaceutical preparations, most notably solid dosage forms such as pills and capsules intended for clinical use. In clinical and pharmaceutical applications, berberine is predominantly administered via the oral route, typically at doses ranging from 0.6 to 2.7 g/day, which aligns with its long-standing use in traditional medicine [110].

### 8.2. Cyclodextrin-Based Formulations

Cyclodextrin-based inclusion complexes represent a promising strategy to improve the solubility and membrane permeability of berberine. Complexation with a cationic γ-cyclodextrin derivative (GCD-PDA) enhanced membrane permeability and altered intracellular distribution, promoting preferential lysosomal accumulation in cancer cells. In murine breast cancer (4T1) and melanoma (B16-F10) cell lines, the cyclodextrin–berberine complex exhibited enhanced cytotoxicity compared to free berberine, with cell death increasing from approximately 25% to 50% in 4T1 cells and from ~50% to ~75% in B16-F10 cells at 131 µM. Importantly, this effect was selective, as no significant toxicity was observed in normal mammary cells (NMuMG) [111].

Cyclodextrin-based inclusion complexes can modulate the interaction of berberine with G-quadruplex DNA structures. Encapsulation of berberine within a modified β-cyclodextrin does not abolish its binding ability but alters the mode and strength of interaction with DNA. Notably, the berberine-loaded cyclodextrin complex exhibits preferential binding to specific G-quadruplex sequences, particularly c-kit (kit22), indicating sequence-dependent variations in binding affinity [112]. Furthermore, cyclodextrin complexation significantly improves the apparent solubility of berberine through host–guest interactions, as evidenced by a linear increase in berberine concentration with increasing β-cyclodextrin levels [113].

### 8.3. Solid Dispersions

Solid dispersion systems represent a promising strategy to overcome the poor bioavailability of berberine. Specifically, an amorphous solid dispersion of berberine with hydrogenated phosphatidylcholine (BHPC-SD) has been shown to significantly improve intestinal permeability, absorption, and systemic bioavailability in rats [114].

More advanced systems, such as berberine–phospholipid complex-based solid dispersions incorporating TPGS and SiO_2_ (BPTS-SD), further enhanced oral absorption through improved lipophilicity and P-gp inhibition, achieving a relative oral bioavailability of up to 322.66% compared to pure berberine, with significant improvements in both Cmax and AUC [115].

In contrast, a pH-sensitive solid dispersion of berberine hydrochloride with Eudragit^®^ S100 demonstrated clear in vitro anticancer activity in human colorectal cancer cell lines (SW480, HCT116, Caco-2), where IC_50_ values decreased from 43.10, 34.13, and 32.82 mM (free berberine) to 28.16, 22.06, and 15.39 mM, respectively, indicating enhanced cytotoxicity and dose-dependent cell death [116].

Furthermore, a spray-dried solid dispersion of berberine (BBR-SD) demonstrated strong in vivo anticancer activity in an A549 orthotopic lung cancer model, reducing tumor volumes by approximately 50% (2-fold decrease vs. untreated controls), along with markedly improved pharmacokinetics (3.46-fold higher Cmax and 6.98-fold higher AUC compared to free berberine) and no significant systemic toxicity [117].

### 8.4. Nanotechnology-Based Formulations

Despite its promising anticancer activity, the clinical application of berberine remains significantly limited by its unfavorable physicochemical and pharmacokinetic properties, including poor aqueous solubility, low gastrointestinal absorption, extensive first-pass metabolism, and consequently very low systemic bioavailability. These limitations result in insufficient drug concentrations at target sites, thereby reducing its therapeutic efficacy in cancer treatment. To address these challenges, nanotechnology-based drug delivery systems have emerged as an effective and versatile strategy to enhance the therapeutic performance of berberine. At the nanoscale, drug carriers can significantly improve solubility, protect the drug from premature degradation, prolong systemic circulation, and enhance cellular uptake. In addition, nanocarriers enable controlled and sustained drug release, which helps maintain effective intracellular drug concentrations over extended periods [117,118].

A wide range of nanocarrier systems have been developed for berberine delivery, including lipid-based nanoparticles, polymeric nanoparticles, dendrimers, mesoporous silica nanoparticles, and biomimetic nanoplatforms. These systems offer distinct advantages such as high drug loading capacity, improved stability, tunable surface properties, and the potential for passive or active targeting to tumor tissues. Notably, advanced nanocarriers can exploit tumor-specific characteristics, such as enhanced permeability and retention (EPR) effect or microenvironment-responsive triggers, to achieve more selective drug accumulation and improved therapeutic outcomes [117,119].

**Figure 4:**
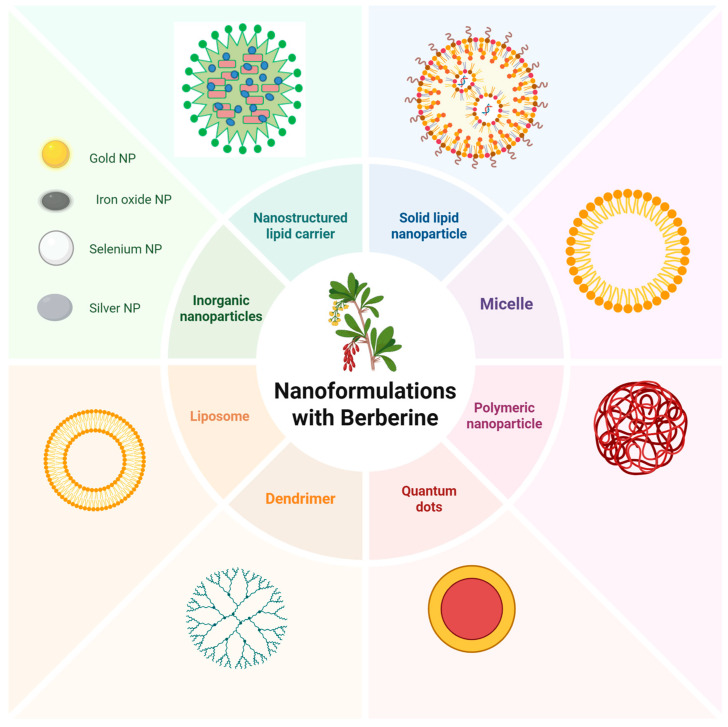
Classification of berberine nano-formulations. “Created in BioRender. Alloush, T. (2026) https://BioRender.com/9lmtqmr, (accessed on 4 April 2026).

Importantly, nanocarrier-based delivery of berberine not only enhances its pharmacokinetic profile but also improves its intracellular bioavailability and biological activity, thereby amplifying its anticancer effects. Consequently, the development of rationally designed berberine-loaded nanocarriers represents a critical approach to overcoming current limitations and advancing its translational potential in cancer therapy. Figure 4 illustrates the major nanoformulation platforms used for berberine delivery.

#### 8.4.1. Liposomal Formulations

Liposomal delivery systems have been widely explored to overcome the poor solubility and bioavailability of berberine and to enhance its anticancer efficacy. In a photodynamic therapy study, berberine-loaded liposomes (Lipo@BBR) were developed and evaluated in A549 spheroid models, where the nanosized liposomal formulation (hydrodynamic diameter 82.7 ± 6.5 nm by DLS) significantly enhanced therapeutic response. The formulation exhibited notable cytotoxicity, with an IC_50_ of 10 ± 0.5 µg/mL under 405 nm laser irradiation, accompanied by increased apoptosis via upregulation of the BAX/BCL2 ratio (6.8-fold increase) and a ~50% reduction in A549 spheroid tumor volume after five PDT fractionation sessions [120].

In another study, glycyrrhetinic acid-modified liposomes co-loaded with berberine and doxorubicin demonstrated enhanced antitumor activity against hepatocellular carcinoma using a co-culture model of Huh-7 and LX-2 cells, as well as in vivo in H22 + NIH-3T3 tumor-bearing mice. These liposomes showed targeted tumor accumulation, improved cellular uptake, and superior inhibition of tumor proliferation, angiogenesis, and extracellular matrix deposition, ultimately leading to significant tumor growth suppression in vivo [121].

Additionally, vitamin C–encapsulated liposomal berberine systems demonstrated selective cytotoxicity toward colon cancer cells such as SW620, where the formulation induced oxidative stress, mitochondrial dysfunction, ATP depletion, and immunogenic cell death (ICD), while enhancing macrophage-mediated phagocytosis of cancer cells, indicating a strong immunotherapeutic potential [122].

Furthermore, targeted berberine liposomes designed for breast cancer stem cells showed the ability to overcome drug resistance by inhibiting ABC transporters and modulating mitochondrial apoptosis pathways in MCF-7 CSCs, with in vivo xenograft studies in nude mice confirming significant therapeutic efficacy and potential for preventing tumor relapse [123].

Overall, liposomal formulations of berberine consistently demonstrate enhanced cytotoxicity, improved cellular uptake, and significant in vivo antitumor activity across multiple cancer models, supporting their role as promising nanocarriers for cancer therapy.

#### 8.4.2. Solid Lipid Nanoparticles (SLNs)

Solid lipid nanoparticles (SLNs) have been developed as an effective delivery platform to overcome the major limitations of berberine, particularly its poor solubility, low bioavailability, and rapid metabolism. In an optimized formulation, berberine hydrochloride-loaded SLNs exhibited a nanoscale particle size of 81.42 ± 8.48 nm, a polydispersity index of 0.25 ± 0.03, and a zeta potential of −28.67 ± 0.71 mV, indicating good stability and homogeneity, along with a high encapsulation efficiency of 70.33 ± 1.53% and sustained drug release over 72 h. Functionally, these physicochemical properties translated into significantly enhanced anticancer activity compared to free berberine. In vitro studies demonstrated that berberine-loaded SLNs more effectively inhibited proliferation of MCF-7, HepG2, and A549 cancer cells in a dose- and time-dependent manner. Notably, after 48 h treatment, the IC_50_ values of berberine-loaded SLNs were markedly reduced to 20.5 μM (MCF-7), 4.8 μM (HepG2), and 15.2 μM (A549), compared to >40 μM, 10.3 μM, and >40 μM for free berberine, respectively, indicating a substantial increase in cellular potency. This enhanced cytotoxicity was further supported by reduced colony formation and significantly higher cellular uptake, reflecting improved intracellular delivery [124].

Among the earliest formulations reported, berberine hydrochloride-loaded solid lipid nanoparticles (BH-SLNs) prepared by the rotary-evaporated film-ultrasonication method demonstrated a mean diameter of 60.5 nm, a zeta potential of 29.7 mV, a drug loading of 8.69%, and a remarkably high entrapment ratio of 97.58%, establishing SLNs as a promising nanocarrier platform for anticancer berberine delivery [125].

#### 8.4.3. Nanostructured Lipid Carriers (NLCs)

Berberine-loaded nanostructured lipid carriers (NLCs) have been investigated as promising nanoplatforms to enhance anticancer efficacy while improving safety profiles. The optimized NLC-B formulation exhibited a particle size of 158.2 ± 1.8 nm, a PDI of 0.305 ± 0.01, a zeta potential of −30.7 ± 0.8 mV, and a remarkably high encapsulation efficiency of 97.7 ± 2.3%. In an in vitro study on gastrointestinal cancers, berberine-loaded NLCs demonstrated significantly enhanced antineoplastic activity in colorectal adenocarcinoma (CaCo-2) cells—with an IC_50_ of 3.35 µM compared to 8.42 µM for free berberine (≈2.5-fold improvement)—and in cholangiocarcinoma (HuCC-T1) cells—with an IC_50_ of 11.86 µM versus 24.66 µM for free berberine (≈2.1-fold improvement). Critically, NLC-B simultaneously reduced cytotoxicity toward non-cancerous systems: viability of normal L929 fibroblasts at 200 µM was 62% (NLC-B) versus 29% (free berberine), and H9c2 cardioblast viability was 47% (NLC-B) versus 20% (free berberine), representing a 2.1–2.35-fold improvement in safety. Moreover, NLC-B reduced liver microsome oxidative stress, as evidenced by a lower MDA increase of 85% compared to 100% with free berberine at 50 µM, indicating reduced hepatotoxic potential. These findings collectively demonstrate a significantly improved therapeutic index for berberine-loaded NLCs compared with the free drug [126].

Mechanistically, NLC formulation improves berberine solubility, bioavailability, and cellular uptake, which are critical limitations of the free drug. In another cancer-related formulation, a dual drug-loaded NLC system containing berberine and doxorubicin for skin cancer showed high entrapment efficiency (~88–89%), improved skin permeation, and deeper tumor deposition, supporting enhanced local delivery and potential reduction of systemic toxicity [127].

The antitumor potential of berberine-loaded NLCs has been further validated in hepatocarcinoma models, berberine-NLCs prepared by hot melting and high-pressure homogenization (particle size 189.3 nm) demonstrated an IC_50_ of 6.3 µg/mL against H22 hepatocarcinoma cells compared to 22.1 µg/mL for bulk berberine (≈3.5-fold improvement), and in vivo studies in H22 solid tumor-bearing mice confirmed a tumor inhibition rate of 68.3% versus 41.4% for bulk berberine at 100 mg/kg (intragastric administration) [128].

#### 8.4.4. PLGA-Based Nanoparticles

Berberine-loaded PLGA nanoparticles have demonstrated significant anticancer potential through both enhanced cellular uptake and improved tumor biodistribution. In an in vitro colorectal cancer model (HCT116 cells), PEG-PLGA–encapsulated berberine (NPBer) showed stronger cytotoxicity compared with free berberine, with reported IC_50_ values of 34.70 μM (NPBer) versus 46.20 μM (free drug), indicating improved antiproliferative efficacy mediated by increased cellular internalization and activation of apoptosis, ferroptosis, and mitochondrial autophagy pathways. This enhanced activity translated into in vivo efficacy in a xenograft mouse model, where NPBer exhibited improved tumor accumulation, sustained retention at the tumor site, and significant inhibition of tumor growth, while histological and biochemical analyses (ALT, AST, creatinine, BUN) suggested no evident systemic toxicity, confirming the biosafety of the PEG-PLGA system [129].

Complementary in vitro evidence in MCF-7 breast cancer cells showed that PLGA–berberine nanoparticles achieved higher cytotoxicity than free berberine, with IC_50_ values of 42.39 μg/mL versus 80.18 μg/mL, alongside controlled drug release and improved encapsulation efficiency, supporting their role in enhancing therapeutic performance [130].

Furthermore, advanced PLGA-based co-delivery systems PDBNP (berberine-loaded PLGA–doxorubicin conjugate nanoparticles) achieved IC_50_ values of 1.94 ± 0.22 μM and 1.02 ± 0.36 μM against MDA-MB-231 and T47D breast cancer cell lines, respectively (MTT assay, 48 h), significantly lower than free doxorubicin (3.89 ± 0.72 μM) or free berberine (4.93 ± 0.34 μM) alone (*p* < 0.05), confirming a synergistic effect. Mechanistically, PDBNP induced necrosis (Annexin V/PI assay), mitochondrial membrane depolarization (JC-1 assay), and sub-G1 cell cycle arrest in MDA-MB-231 cells. In vivo pharmacokinetics in Sprague Dawley rats revealed a ≈14.65-fold increase in doxorubicin half-life (t½: 42.51 ± 3.47 h vs. 2.90 ± 0.28 h for free drug) with an ≈18.8-fold increase in AUC_0–_∞, alongside a 5.52-fold increase in berberine half-life (25.29 ± 4.01 vs. 4.58 ± 0.39 h). Safety evaluation confirmed substantially reduced hemolytic toxicity (PDBNP 5.23% vs. doxorubicin 26.47%; *p* < 0.0001; ≅5× safer) and negligible cytotoxicity toward normal hPBMCs, collectively confirming the enhanced safety and efficacy profile of PLGA-based co-delivery in combination cancer therapy [131].

#### 8.4.5. Chitosan-Based Nanoparticles

Berberine-loaded chitosan nanoparticles (BBR-COSNPs) have been investigated as a promising oral anticancer delivery system, overcoming berberine’s inherently poor aqueous solubility and gastrointestinal bioavailability via EPR-mediated mucosal absorption. In an in vivo urethane-induced lung cancer model (male albino mice, urethane 1 mg/g i.p. ×3), oral administration of BBR-COSNPs at 75 mg/kg/day for 10 weeks significantly suppressed tumor progression compared with free berberine: oxidative stress markers were reduced—MDA (233.00 → 140.00 nmol/g) and NO (4.76 → 2.55 μmol/g)—while antioxidant defenses were restored—GSH (2.40 → 4.63 μmol/g) and SOD (141.70 → 202.50 U/g). Inflammatory signaling was suppressed via significant reductions in serum NF-κB and COX-2 immunostaining (reduced from diffuse to pale brown staining). Tumor angiogenesis was inhibited through decreased serum VEGFR2 (11.87 → 6.30 ng/mL) and lung HIF-1α mRNA (3.66 → 2.22 Rq). Apoptosis was promoted via significantly elevated lung caspase-9 mRNA (0.77 → 3.01 Rq) and serum BAX (95.30 → 203.25 pg/mL). Safety assessment demonstrated zero mortality in the BBR-COSNPs group versus two deaths in the free BBR group, with significantly lower ALT, AST, urea, and creatinine levels, collectively confirming a markedly improved safety and therapeutic profile of the chitosan nanoparticle delivery system compared with free berberine [132].

Complementary in vitro evidence using chitosan-based nanoplatforms further supports the anticancer potential: for example, berberine-decorated chitosan nanoparticles (CS-ZnO-Ber NPs) exhibited strong cytotoxicity against MCF-7 breast cancer cells with an IC_50_ of 7.41 µg/mL, inducing apoptosis, cell cycle arrest (sub-G1 phase), and inhibition of cell migration, while showing acceptable hemocompatibility and reduced toxicity toward normal HEK-293 cells [133].

Additionally, other studies confirm that chitosan nanoparticles enhance berberine stability, controlled release, and systemic exposure (e.g., increased Cmax and ~122% relative bioavailability in rats), which supports their role in overcoming berberine’s intrinsic limitations such as poor solubility and rapid elimination [134].

Overall, these findings indicate that chitosan-based nanocarriers not only potentiate the anticancer activity of berberine through improved delivery and cellular uptake but also contribute to reduced systemic toxicity and enhanced therapeutic index.

#### 8.4.6. PEG-Functionalized Nanoparticles

PEGylated mixed micelles composed of PEG–phosphatidylethanolamine (PEG-PE) and d-α-tocopheryl polyethylene glycol 1000 succinate (TPGS) have been developed to enhance the delivery and anticancer performance of berberine by overcoming its poor solubility and bioavailability. This nanocarrier system markedly improves aqueous solubilization (≈300% increase), stability, and pharmacokinetic behavior, while enabling sustained drug release and efficient tumor accumulation. In vitro, these PEG-based micelles demonstrated significantly enhanced cytotoxicity against prostate cancer cell lines (PC3 and LNCaP), achieving up to 16–18-fold lower IC_50_ values compared to free berberine. he improved anticancer activity is attributed to enhanced cellular uptake, mitochondrial-mediated apoptosis, and the ability of TPGS to inhibit P-glycoprotein–mediated drug efflux, thereby helping to overcome multidrug resistance. Additionally, PEGylation provides a “stealth” effect that prolongs systemic circulation and facilitates passive tumor targeting via the enhanced permeability and retention (EPR) effect, while maintaining relatively low systemic toxicity [135].

In another PEG-based approach, Wang et al. designed biotin (BI)-modified nanomicelles (BBR@PTX NPs) by chemically grafting paclitaxel (PTX) onto a BI-PEG carrier and physically encapsulating berberine (BBR) at a synergistic PTX:BBR ratio of 1:10, yielding nanoparticles of 140 ± 1.02 nm with narrow PDI (0.1–0.2), drug loadings of 9.5% (PTX) and 28.06% (BBR), and encapsulation efficiencies of 8.6% and 33.5%, respectively. In MDA-MB-231 triple-negative breast cancer (TNBC) cells, BBR@PTX NPs achieved a synergy score of 23.874 (SynergyFinder 3.0), inducing apoptosis in 42.3 ± 1.78% of cells, significantly surpassing free BBR (18.77 ± 0.89%) and free PTX (22.45 ± 1.03%) while also demonstrating the strongest inhibition of cellular proliferation (colony formation assay), migration, and invasion (Transwell system) among all treatment groups. The biotin targeting moiety enabled receptor-mediated endocytosis, enhanced lysosomal escape by 6 h, and achieved macrophage immune-escape via PEGylation. In vivo, BBR@PTX NPs markedly suppressed xenograft tumor growth (4–5-fold expansion vs. ~15-fold in controls), with IVIS fluorescence imaging confirming selective tumor accumulation from 8 h (peak 24 h), and histological analysis (H&E, Ki-67, Bcl-2, TUNEL) confirming anti-proliferative and pro-apoptotic activity alongside an absence of significant organ toxicity, establishing superior biosafety and therapeutic efficacy compared with free-drug formulations [136].

#### 8.4.7. Gold Nanoparticles (AuNPs)

Berberine has been effectively integrated with gold nanoparticle-based delivery systems to enhance its anticancer activity, cellular uptake, and therapeutic performance. A liposomal nanocomplex co-loaded with berberine and citrate gold nanoparticles (Lipo@AuNPs@BBR) was developed for photodynamic therapy against A549 spheroid cells, exhibiting a nanoscale size and enhanced phototoxic effects through reactive oxygen species (ROS) generation. The system demonstrated significant cytotoxicity, with a dark-toxicity IC_50_ of 80 µg/mL; when combined with 405 nm laser irradiation (15 J cm^−2^), the combination IC_50_ was reduced to 60 µg/mL, and at 80 µg/mL with laser, spheroid cell viability fell to ~34%, highlighting the synergistic effect of gold nanoparticles and berberine in 3D tumor models [137].

Similarly, gold nanoparticle–based nanocarriers such as gold–collagen–berberine (Au–Col–BB) were shown to enhance intracellular delivery and anticancer efficacy in brain tumor models using DBTRG cells. These nanoparticles significantly increased apoptosis and reduced proliferation by promoting cell cycle arrest and enhancing cellular uptake via endocytosis pathways. In vivo studies in BALB/c mice further confirmed efficient accumulation in brain tissue and favorable safety profiles, demonstrating the potential of gold nanoparticle-mediated delivery to overcome berberine’s bioavailability limitations [138,139].

In another approach, berberine was incorporated into ascorbic acid-stabilized gold nanoparticles in combination with letrozole (LTZ-BBR@AA-AuNPs), achieving controlled drug release and strong cytotoxic activity against MDA-MB-231 cells, with an IC_50_ of 2.04 ± 0.011 µg/mL after 48 h, indicating potent anticancer efficacy [140].

Overall, gold nanoparticle-based systems markedly improve berberine delivery by increasing cellular uptake, enabling synergistic therapeutic mechanisms (e.g., ROS-mediated damage), and enhancing both in vitro cytotoxicity and in vivo antitumor efficacy across multiple cancer models.

#### 8.4.8. Iron Oxide Nanoparticles (IONPs)

Berberine loaded iron oxide nanoparticles have been explored as targeted anticancer systems, particularly for improving tumor-specific delivery and overcoming bioavailability limitations. In a mouse solid tumor model, NP-BBN-SAN complexes demonstrated the highest antitumor efficacy, reducing tumor volume from an initial 1 cm^3^ to 0.178 ± 0.01 cm^3^ by day 12 (*p* < 0.001), compared with progressive growth to 1.57 ± 0.08 cm^3^ in untreated controls. The treatment induced extensive cellular DNA damage (comet assay; *p* < 0.05) and significantly downregulated HIF-1α (*p* < 0.001), VEGF (*p* < 0.001), AKT, and BCL-2, while upregulating BAX and caspase-8 via the TNF-α extrinsic apoptotic pathway. Histopathologically, tumor tissues showed condensed nuclei indicative of apoptosis, while liver and kidney remained morphologically normal. Serum creatinine and SGOT in NP-BBN-SAN-treated mice were 0.637 ± 0.07 mg/dL and 171.67 ± 1.2 U/L, respectively—non-significantly different from normal animals, confirming limited systemic toxicity. Survival analysis demonstrated 100% survival at 30 days in the NP-BBN-SAN group, versus <20% in untreated controls [141].

In contrast, other iron oxide nanoparticle systems derived from Berberis vulgaris extracts (rich in berberine) showed in vitro cytotoxicity against breast cancer cell lines (MCF-7 and MDA-MB-231) via apoptosis induction and oxidative stress mechanisms, with IC_50_ values of 21.9 µg/mL and 27.17 µg/mL for MCF-7 and MDA-MB-231, respectively (at 24 h), and minimal toxicity toward normal Vero cells, confirming cancer-selective activity [142].

#### 8.4.9. Selenium Nanoparticles

Berberine-based selenium nanoparticles (Ber-SeNPs) have been investigated as promising anticancer agents, demonstrating markedly enhanced efficacy compared to free berberine. In an in vitro study on HepG2 hepatocellular carcinoma cells, Ber-SeNPs exhibited extremely potent cytotoxicity with an IC_50_ of 0.04 µg/mL approximately 667-fold lower than free berberine (26.69 µg/mL) and lower than cisplatin (0.33 µg/mL) with apoptosis induction via the intrinsic mitochondrial pathway, G_1_ cell-cycle arrest, and elevated oxidative stress markers, while showing greater selectivity toward cancer cells over normal BNL liver cells (IC_50_ > 100 µg/mL for free Ber; 1.02 µg/mL for Ber-SeNPs) [143].

Complementing this, an in vivo study using Ehrlich solid tumor (EST)-bearing mice showed that Ber-SeNPs (0.5 mg/kg) significantly reduced tumor size and volume, improved survival rate, and alleviated oxidative stress, while promoting apoptosis via modulation of Bcl-2/Bax and caspase-3 pathways. Additionally, treated animals exhibited improved histopathological features and prolonged lifespan compared to untreated controls, with indications of lower systemic toxicity compared to conventional chemotherapy (cisplatin) [144].

Overall, these findings confirm that berberine-selenium nanoparticles enhance anticancer efficacy through synergistic mechanisms involving apoptosis induction, oxidative stress modulation, and improved bioavailability, while maintaining relatively favorable safety profiles.

#### 8.4.10. Quantum Dot

Berberine has been successfully incorporated into quantum dot–like nanomaterials, particularly carbon dots (CDs), to overcome its inherent limitations such as poor solubility and low bioavailability. Berberine-derived carbon dots (Ber–CDs), synthesized via hydrothermal approaches, retain the intrinsic pharmacological activity of the parent compound while exhibiting improved physicochemical properties, including enhanced water solubility, photostability, and cellular uptake. These nanostructures demonstrate significant anticancer activity, showing dose-dependent cytotoxic effects across multiple cancer cell lines, including HepG2 and SMMC-7721 (liver cancer), A549 (lung cancer), MCF-7 (breast cancer), and H22 (mouse liver cancer), while exhibiting comparatively lower toxicity toward normal cells such as HL-7702 and HUVEC. In vivo (H22 model, 50 mg/kg i.v. × 14 days), Ber-CDs significantly reduced tumor volume and weight without systemic toxicity confirmed by unchanged body weight, normal serum biochemistry (ALT, AST, BUN, CRE, TG, ALB, ALP, TC), and intact organ histopathology (heart, liver, spleen, kidney, lung) [145].

More recently, Feng et al. (2025) developed BSA-modified carbon dots loaded with berberine (BSA@BBR@CDs, 13.1 nm) to overcome berberine’s poor bioavailability (<5% in humans), achieving superior cytotoxicity in 4T1 breast cancer and B16 melanoma cells compared to free berberine [146]. In vivo, intravenous administration (5 mg/kg) in 4T1 tumor-bearing mice induced significant tumor shrinkage and prolonged survival via mitochondrial membrane potential disruption, elevated intracellular ROS, and apoptosis activation (Bak1, Cleaved Caspase-3, Granzyme B), without systemic toxicity or immune suppression [146].

#### 8.4.11. Dendrimer

Berberine has been successfully incorporated into polyamidoamine (PAMAM) dendrimer systems using both encapsulation and covalent conjugation strategies to overcome its poor bioavailability and enhance anticancer efficacy. In one study, G4 PAMAM dendrimers were used to deliver berberine either as an encapsulated system (BPE: berberine-loaded PAMAM dendrimer via physical encapsulation) or as a conjugated nanoformulation (BPC: berberine–PAMAM conjugate formed through covalent bonding), showing nanometric sizes (~180–210 nm) and sustained drug release (up to ~98% within 24 h). Cytotoxicity evaluated by MTT assay demonstrated significantly enhanced anticancer activity of the conjugated dendrimer system against human breast cancer cell lines MCF-7 and MDA-MB-468 compared to free berberine, while maintaining low hemolytic toxicity (<5%) and good biocompatibility. Importantly, in vivo studies in albino rats revealed markedly improved pharmacokinetics, with the half-life increasing to 14.33 h versus 6.7 h for free berberine, along with enhanced AUC, indicating improved systemic exposure [147].

In another dendrimer-based system, berberine was co-delivered with methotrexate (MTX) using G4 PAMAM (MTX-PAMAM-BER, 163.1 ± 5.67 nm, BER loading ~54%) to target HeLa cervical cancer cells via RFC1 folate receptor-mediated uptake. The nanocomposite achieved an IC_50_ of 25 µg/mL (48 h)—6-fold lower than free berberine (150 µg/mL) and 4-fold lower than MTX-PAMAM alone (100 µg/mL)—and induced 60.7% apoptosis (Annexin V/PI) with the highest intracellular ROS generation among all tested groups [148].

Additionally, berberine-loaded PEGylated G4 PAMAM dendrimers (186 nm, EE ~70%, zeta −21.8 mV) demonstrated markedly improved cellular uptake in AMJ-13 and BT-20 cancer cell lines (fluorescence microscopy) and significantly enhanced cytotoxicity against MCF-7 breast cancer cells (IC_50_ = 10.8 µM vs. >50 µM for free berberine; >4.6-fold improvement), with sustained drug release of 70.23% at 72 h [149].

The nanoformulations presented in Table 1 (refs. [120,121,122,123,124,125,126,127,128,129,130,131,132,133,134,135,136,137,138,139,140,141,142,143,144,145,146,147,148]) encompass seven major carrier classes: liposomes, solid lipid nanoparticles (SLNs), nanostructured lipid carriers (NLCs), polymeric nanoparticles (PLGA/PEG-PLGA), chitosan nanoparticles, polymeric micelles, metallic nanoparticles (gold, iron oxide, selenium), carbon dots, and PAMAM dendrimers, collectively demonstrating that nanoencapsulation consistently enhances berberine’s aqueous solubility, cellular uptake, and anticancer potency relative to the free drug. Among lipid-based systems, liposomes achieved the highest encapsulation efficiencies (EE% 89%) and enabled the most mechanistically diverse strategies: Lipo@BBR [120] exploits BBR’s intrinsic photosensitiser activity for PDT, reducing A549 spheroid volume by ~50%; the GA-modified liposome [121] achieves liver-selective ASGPR targeting with an in vivo tumour growth inhibition of 90.82%; and the Vitamin C liposome [122] uniquely induces immunogenic cell death (ICD) and synergises with anti-CD47 immunotherapy. SLNs [125] record the highest EE% in table (97.58%) and prioritise oral bioavailability, while NLCs [126,127,128] balance high EE% (88–98%) with active targeting, as exemplified by the mannose-conjugated NLC [127] penetrating 69.9 µm into skin tumours. Among polymeric systems, PEG-PLGA nanoparticles [129] uniquely activate ferroptosis, mitophagy, and autophagy simultaneously (RNA-Seq-validated) and achieve the highest tumour accumulation by IVIS imaging, whereas dual-drug PLGA-PDBNP [131] prolongs plasma half-life 14.6-fold and reduces haemolysis from 26.47% (free DOX) to 5.23%. Chitosan nanoparticles [132,133,134] exploit cationic charge for mucoadhesion; BBR-COSNPs [132] achieved zero mortality in an in vivo lung-cancer model versus three deaths with free BBR, and CS-ZnO-Ber [133] demonstrated a 3.1× cancer-selectivity window (IC_50_ 7.41 vs. 23 µg/mL in normal cells). Polymeric micelles offer the largest IC_50_ fold-improvements: PEG-PE/TPGS micelles [135] achieve an 18-fold reduction in prostate cancer IC_50_ with simultaneous P-gp bypass, and biotin-targeted nanomicelles co-loading BBR + PTX [136] record the highest synergy score in this table (23.87) with the strongest relative in vivo suppression in TNBC. Gold nanoparticles [137,138,139,140] provide multimodal platforms combining photothermal, photodynamic, and active-targeting modalities; Au–Col–BB [138] records one of the lowest IC_50_ values in the table (1 µg/mL in glioblastoma), while LTZ-BBR@AA-AuNPs [140] demonstrate pH-responsive synergistic delivery of letrozole + BBR in TNBC. Inorganic selenium nanoparticles [143,144] achieve the most striking potency gains: Ber-SeNPs [143] record an IC_50_ of 0.04 µg/mL in HepG2 hepatocarcinoma—667-fold lower than free BBR—with a ~25× cancer/normal selectivity window, and SeNPs-Ber [144] surpasses cisplatin in vivo at one-tenth the dose (0.5 vs. 5 mg/kg, oral). Iron oxide NP-BBN-SAN [141] is unique in achieving 100% animal survival at 30 days through hypoxia-targeted delivery via sanazole. Carbon dots [145,146] are the smallest carriers (2–15 nm) and the only theranostic platforms: Ber-CDs [145] uses BBR as the carbon precursor for intrinsically fluorescent dots enabling simultaneous imaging and therapy, while BSA@BBR@CDs [146] adds pH/enzyme-responsive release and confirmed mitochondrial targeting in vivo. Finally, PAMAM dendrimers [147,148] enable precise molecular engineering: G4-PAMAM-BBR [147] doubles plasma half-life (14.33 vs. 6.7 h), and MTX-PAMAM-BER [148] employs methotrexate as a dual folate-receptor ligand and DHFR inhibitor, yielding a 6-fold IC_50_ reduction with 60.7% apoptosis in HeLa cervical cancer. Taken together, these findings underscore that the choice of carrier should be guided by the therapeutic objective: SLNs and chitosan NPs for oral bioavailability, micelles and PLGA NPs for MDR reversal and mechanistic breadth, selenium and gold NPs for maximum cytotoxic potency, liposomes for combination immunotherapy or PDT, carbon dots for theranostic applications, and PAMAM dendrimers for receptor-targeted co-delivery.

## 9. Limitations and Translational Roadmap for Berberine

Natural products derived from traditional medicine, including compounds such as berberine, often face significant limitations such as poor aqueous solubility, low bioavailability, and inconsistent pharmacokinetic profiles, which hinder their clinical translation [150].

Despite these preclinical advances, clinical evidence for berberine in oncology remains limited. An umbrella review encompassing 11 meta-analyses and up to 4985 participants confirmed that, while berberine significantly reduced colorectal adenoma recurrence at one year (RR = 0.77, 95% CI: 0.64–0.92) and two years (RR = 0.82, 95% CI: 0.74–0.90)—the only cancer-related outcome supported by moderate-quality GRADE evidence—the vast majority of berberine-related clinical outcomes (72.92%) were rated as low to very low quality [151].

This is further corroborated by a broader overview of 54 systematic reviews encompassing 452 assessed outcomes, in which 69.03% were rated as very low quality by GRADE; notably, despite demonstrated efficacy across nine chronic conditions, berberine remains conspicuously absent from clinical practice guidelines, primarily due to methodological inconsistencies, small sample sizes, and the geographic homogeneity of trial populations toward Asian participants [152].

Regulatory considerations represent a critical aspect in the development of berberine as an adjuvant in cancer therapy. Currently, berberine is widely used in plant preparations and dietary supplements rather than as an approved pharmaceutical agent, and its safety is evaluated within food regulatory frameworks such as Regulation (EC) No 1925/2006. The European Food Safety Authority (EFSA) has highlighted concerns regarding adverse effects and potential drug interactions associated with berberine-containing products, emphasizing the need for rigorous safety assessment prior to its use in humans [153].

Furthermore, increasing use of berberine in nutraceutical formulations raises additional regulatory challenges related to product quality and safety. Recent studies have reported that synthetic production routes may introduce potentially hazardous impurities, including nitrosamines, which are classified as genotoxic compounds and are strictly regulated under international guidelines such as ICH M7. In addition, residual solvents and chemical intermediates used during synthesis are subject to regulatory limits defined by ICH Q3C due to their toxicological risks [154].

These findings indicate that despite its natural origin, berberine requires comprehensive regulatory evaluation encompassing safety, quality control, and manufacturing standards before it can be reliably translated into clinical combination therapies.

## 10. Conclusions

Berberine (BBR) has emerged as a scientifically compelling multifunctional adjuvant in cancer therapy, distinguished by a pleiotropic pharmacological profile that converges on the modulation of apoptosis, cell-cycle regulation, and multiple oncogenic signalling networks. The BCL-2/BAX axis consistently emerges as a central node of its antitumour activity across diverse cancer types, operating in concert with downstream effectors of the PI3K/Akt/mTOR, AMPK, and NF-κB pathways to suppress tumour survival, proliferation, and metastasis. Critically, BBR functions not merely as a standalone cytotoxic agent but as a chemosensitiser capable of potentiating the activity of doxorubicin, cisplatin, tamoxifen, 5-fluorouracil, gemcitabine, and targeted agents such as EGFR-TKIs. However, BBR’s intrinsic pharmacokinetic liabilities, low oral bioavailability, extensive first-pass metabolism, and rapid systemic clearance have historically constrained its therapeutic translation. The nanotechnological strategies reviewed herein represent a decisive response to these limitations. Nanoencapsulation enhances aqueous solubility, prolongs circulation half-life, enables active or passive tumour targeting, and achieves IC_50_ reductions of up to several hundred-fold relative to the free drug. Focusing on lung cancer, several nanoformulation strategies reviewed herein have demonstrated compelling efficacy: berberine-loaded solid lipid nanoparticles (SLNs) reduced the IC_50_ in A549 non-small cell lung cancer cells to 15.2 μM compared with >40 μM for the free drug; chitosan-based nanoparticles (BBR-COSNPs) suppressed tumour progression and attenuated NF-κB/COX-2-mediated inflammation in an in vivo urethane-induced lung cancer model; and a liposomal nanocomplex co-loaded with gold nanoparticles (Lipo@AuNPs@BBR) significantly enhanced photodynamic-mediated cytotoxicity in A549 spheroids, collectively highlighting solid lipid and chitosan-based nanoplatforms as particularly promising nanocarriers for pulmonary oncological applications. The safety profile of BBR warrants nuanced interpretation. Its low oral bioavailability, paradoxically, contributes to its favourable gastrointestinal tolerability, whereas parenteral administration carries meaningful cardiotoxic and haematological risks that must inform clinical protocol design. Addressing these gaps will require harmonised preclinical-to-clinical dosing frameworks and investment in scalable, GMP-compliant nanoformulation manufacturing. Taken together, the mechanistic depth, combination versatility, nanotechnological adaptability, and emerging clinical signals presented in this review collectively substantiate BBR’s candidacy as a transformative adjuvant in oncology, one whose full therapeutic potential remains contingent on the rigorous clinical evidence that the field has yet to generate.

## Figures and Tables

**Table 1:** Comparison of Berberine Nanoformulations: Composition, Physicochemical Properties, Target Cancer, and Key Anticancer Findings.

Formulation Type	Drug Content (BBR/Combination)	Particle Size (nm)	Target Cancer	Key Anticancer Findings	Ref.
Liposome (Lipo@BBR)	BBR alone	82.7 ± 6.5 nm (DLS) 56.99 ± 3.74 nm (TEM/SEM)	Lung cancer (A549 spheroids)	EE% = 89 ± 2%; ZP = −10.7 mV; BBR as photosensitizer (418 nm); PDT (405 nm, 25 J/cm^2^): IC_50_ = 10 µg/mL (A549 spheroids); spheroid vol ↓~50%; BAX/BCL2 = 6.8; ↑ROS → mitochondrial apoptosis.	[120]
Liposome (GA-modified, BBR + DOX)	BBR + Doxorubicin	124.46 ± 0.89 nm (DLS)	Hepatocellular carcinoma (Huh-7/LX-2)	EE%: DOX 72.56%, BBR 59.39%; ZP = −1.48 mV; PDI = 0.104; IC_50_ = 0.684 µg/mL (≈2.8× vs. free DOX); GA/ASGPR targeting; TGI = 90.82% in vivo; ↓ECM, ↓angiogenesis; low systemic toxicity.	[121]
Liposome (Vit. C-BBR)	BBR + Vitamin C	108.8 nm (DLS) (before loading: 108.1 nm) PDI = 0.032	Colon cancer (LS180, SW620) Normal: CCD112CoN	EE% >90%; PDI = 0.032; IC_50_ (72 h): SW620 = 5 µM, LS180 = 11 µM; normal CCD112CoN = 144 µM (selective); ICD: ↑ROS, ↑CRT, ↑HMGB1 (4×); ↑macrophage phagocytosis 3×; synergy with anti-CD47.	[122]
Liposome (Targeted, BBR)	BBR alone	N/R	Breast cancer (MCF-7 CSCs)	Overcame MDR via ABC transporter inhibition; mitochondrial apoptosis pathway activated; confirmed in vivo (xenograft, nude mice).	[123]
Solid Lipid NP (SLN-BBR)	BBR alone	~100 (optimised)	Breast cancer (MCF-7)	↑cytotoxicity & cellular uptake vs. free BBR; improved encapsulation efficiency & oral bioavailability.	[124]
Solid Lipid NP (BH-SLN)	BBR·HCl alone	60.5 nm (mean diameter)	General/hepatic	EE% = 97.58%; DL = 8.69%; ZP = +29.7 mV; improved solubility & oral bioavailability of BBR·HCl.	[125]
NLC-B	BBR alone	158.2 ± 1.8	Gastrointestinal tumors (CaCo-2 colorectal; HuCC-T1 cholangiocarcinoma)	EE% = 97.7%; ZP = −30.7 mV; PDI = 0.305; IC_50_ = 3.35 µM vs. 8.42 µM (free BBR, CaCo-2, 2.5×); IC_50_ = 11.86 µM vs. 24.66 µM (HuCC-T1); ↓toxicity to normal cells.	[126]
NLC (BBR + DOX, mannose-conj.)	BBR + Doxorubicin	202.7 ± 0.9	Skin cancer (Melanoma, SCC A431)	EE%: DOX 89.5%, BBR 88.5%; ZP = −23.7 mV; mannose receptor-mediated targeting; skin penetration 69.9 µm; in silico: ↑affinity for DNA-Topo-II & PI3K.	[127]
NLC-BBR (H22 model)	BBR alone	189.3	Hepatocarcinoma (H22)	IC_50_ = 6.3 µg/mL vs. 22.1 µg/mL (free BBR, ≈3.5×); significant tumor suppression in vivo (H22 model).	[128]
PEG-PLGA NP (NPBer)	BBR alone	102.3 ± 1.9 nm (DLS)	Colorectal cancer (HCT116)	EE% = 48.9%; DLC = 4.9%; IC_50_ (HCT116, 36 h) = 34.70 µM vs. 46.20 µM; ↑ferroptosis, ↑mitophagy, ↑autophagy; ↓Wnt, ↓Hippo, ↓Notch; highest tumor accumulation (IVIS) & growth inhibition in vivo; ↑TUNEL, ↓Ki-67.	[129]
PLGA NP (BBR)	BBR alone	234 ± 2.48 nm (DLS)	Breast cancer (MCF-7)	EE% = 84.2%; ZP = −8.21 mV; PDI = 0.324; IC_50_ (MCF-7, 48 h) = 42.39 µg/mL vs. 80.18 µg/mL (≈1.9×); biphasic release 61.46% at 48 h (pH 5.2); safe on HFF & MCF-10A normal cells.	[130]
PLGA NP (PDBNP, BBR + DOX)	BBR + Doxorubicin	198.01 ± 3.25	Breast cancer (MDA-MB-231, T47D)	EE% = 52.98%; ZP = −8.76 mV; IC_50_ = 1.94 µM (MDA-MB-231), 1.02 µM (T47D); hemolysis 5.23% vs. 26.47% (free DOX); t½ ↑14.6× in vivo.	[131]
Chitosan NP (BBR-COSNPs)	BBR alone	45 ± 5.6	Lung cancer (urethane-induced, in vivo)	ZP = +39.82 mV; zero mortality vs. 3 deaths (free BBR group); NF-κB/COX-2↓; BAX/Caspase-9↑; VEGFR2/HIF-1α↓; hepato- & renoprotective.	[132]
Chitosan NP (CS-ZnO-Ber)	BBR + ZnO	34.71–407.7 (range)	Breast cancer (MCF-7)	ZP = −38.5 mV; IC_50_ = 7.41 µg/mL (MCF-7) vs. 23 µg/mL (normal HEK-293, 3.1× selective); ROS↑ 85%; apoptosis 43%; migration↓; hemolysis <5%.	[133]
Chitosan NP (BBR)	BBR alone	406 ± 25	General/oral bioavailability (Wistar rats)	EE% = 31.5%; ZP = +41.6 mV; relative oral bioavailability = 122.06%; AUC_0–24_↑ 22%; improved GI stability; stable 6 months at 5 °C.	[134]
PEG-PE/TPGS Mixed Micelles (Brb-mMic)	BBR alone	24.1 ± 1.8	Prostate cancer (PC3, LNCaP spheroids)	EE% = 95.7%; ZP = −23.5 mV; IC_50_ = 4.86 µM (PC3) vs. 87 µM (free BBR, 18×); P-gp bypass (TPGS); oral bioavailability 5% →15%; hemolysis <5%.	[135]
Biotin-modified Nanomicelle (BBR@PTX NPs)	BBR + Paclitaxel	140 ± 1.02	Triple-Negative Breast Cancer (MDA-MB-231)	EE%: BBR 33.5%, PTX 8.6%; synergy score = 23.87 (BBR:PTX 10:1); apoptosis↑ 42.3%; biotin receptor targeting; in vivo: tumor vol↑ 4× vs. 15× (control); no organ damage.	[136]
Gold NP (Lipo@AuNPs@BBR)	BBR + AuNPs	392.1 (DLS) 100 (TEM)	Lung cancer (A549, 3D)	EE% = 14% (BBR); dark IC_50_ = 80 µg/mL → laser IC_50_ = 60 µg/mL (405 nm, 15 J/cm^2^); viability↓ to 34.12%; LDH↑ 2.92×; synergistic ROS (PDT on 3D spheroids).	[137]
Gold NP (Au–Col–BB)	BBR + Collagen-AuNPs	287 (DLS) 250 (SEM)	Glioblastoma (GBM) (DBTRG-05MG)	IC_50_ = 1 µg/mL (DBTRG, 48 h); no toxicity to normal BAECs; apoptosis↑ 4.8×; sub-G1 ↑ 19.4%; migration↓ (MMP-2↓); ROS↓; TNF-α/IL-1β↓.	[138]
Gold NP (Au–Col–BB) [Her-2]	BBR + Collagen-AuNPs	227	Breast cancer (Her-2)	Viability↓ to 0.22× (10 µg/mL); apoptosis 80.6% (Her-2+ cells); MMP-2↓; Bax↑, Bcl-2↓; in vivo tumor weight↓; selective vs. normal BAECs.	[139]
Gold NP (LTZ-BBR@AA-AuNPs)	BBR + Letrozole	81.23 ± 4.0	Breast cancer (TNBC, MDA-MB-231)	EE%: LTZ 58%, BBR 54%; IC_50_ = 2.04 µg/mL (MDA-MB-231, 48 h); pH-responsive release (72% at pH 5.0); ROS↑ 3.06×; synergistic LTZ + BBR cytotoxicity.	[140]
Iron Oxide NP (NP-BBN-SAN)	BBR alone	144.44 ± 94.9 (DLS) 13.97 (XRD)	Solid tumor (Dalton Lymphoma, Swiss albino mice, in vivo)	100% survival at 30 days; tumor vol↓ to 0.4 cm^3^ vs. 1 cm^3^ (control); HIF-1α/VEGF/AKT/BCL-2↓; BAX/Caspase-8/TNF-α↑; hypoxia-targeted via Sanazole.	[141]
Iron Oxide NP (PF127-Fe_2_O_3_)	BBR (from B. vulgaris extract)	174.90 (DLS) 35–55 (SEM/TEM) 32.54 (XRD)	Breast cancer (MCF-7, MDA-MB-231) vs. Vero (normal)	Green synthesis (B. vulgaris extract + Pluronic F-127); IC_50_ (MCF-7, 24 h) = 21.9 µg/mL; (MDA-MB-231) = 27.17 µg/mL; ROS-mediated apoptosis; low toxicity to normal Vero cells.	[142]
Selenium NP (Ber-SeNPs)	BBR + Se	171.5 ± 4.2 nm (DLS)	Hepatocellular carcinoma (HepG2)	ZP = −12.4 mV; IC_50_ (HepG2) = 0.04 µg/mL (667× lower than free BBR); selective: normal BNL IC_50_ = 1.02 µg/mL; ↑p53/Bax/Caspase-3, ↓Bcl-2; in vivo (Ehrlich): tumor↓, survival↑.	[143]
Selenium NP (SeNPs-Ber)	BBR (as reducing agent) + Se	113.4 nm (DLS) <200 nm (TEM)	Solid tumor (Ehrlich Ascites Tumor, in vivo Swiss albino mice)	ZP = −29.1 mV; green synthesis; oral 0.5 mg/kg → tumor↓ vs. cisplatin 5 mg/kg; ↑Bax/Caspase-3, ↓ Bcl-2; ↓ MDA, ↓NO, ↑GSH; ↑survival rate.	[144]
Carbon Dots (Ber-CDs)	BBR (as carbon source)	11.5 nm (DLS) 2–5 nm (TEM/HRTEM)	Multiple cancer lines: HepG2, SMMC-7721, A549, MCF-7, H22 (in vivo)	Selective IC_50_ (cancer ≪ normal cells); bioimaging via CLSM (HepG2); in vivo (H22): tumor↓, EPR accumulation; theranostic platform (imaging + therapy).	[145]
Carbon Dots (BSA@BBR@CDs)	BBR + CDs + BSA	13.1 ± 2.3 nm (TEM) CDs core: 4.7 ± 0.4 nm	Breast cancer (4T1) Melanoma (B16) (in vivo 4T1 xenograft)	pH-responsive release (↑acidic pH + Proteinase K); BBR:CDs = 1:1; ↑cytotoxicity vs. free BBR (4T1, B16); mitochondria targeting; ↓Δψm, ↑ROS, ↑Bak1 → apoptosis; in vivo: tumor shrinkage, ↑survival.	[146]
PAMAM Dendrimer (G4-PAMAM-BBR) BPC & BPE	BBR (conjugated BPC + encapsulated BPE)	BPC: 210.7 ± 9.98 nm BPE: 180.1 ± 5.90 nm (DLS)	Breast cancer (MCF-7, MDA-MB-468)	EE% = 29.9% (BPE); drug conjugation = 37.49% (BPC, ≈24 BBR/PAMAM); anticancer: BPC > BPE > free BBR (MCF-7, MDA-MB-468); t½ BPC = 14.33 h vs. BBR 6.7 h (↑2.1×); hemolysis < 5%.	[147]
PAMAM Dendrimer (MTX-PAMAM-BER)	BBR + Methotrexate	163.10 ± 5.67 nm (DLS)	Cervical cancer (HeLa)	EE% ≈54%; ZP = +1.75 mV; PDI = 0.428; pH-responsive release (pH 5.4: 47%); IC_50_ (HeLa, 48 h) = 25 µg/mL (≈6× lower than free BBR); folate-receptor targeting (MTX/RFC1); apoptosis 60.7%; ↑ROS.	[148]

## Data Availability

No new data were created or analyzed in this study. Data sharing is not applicable to this article.

## Abbreviations

BBR	Berberine
MDR	Multidrug resistance
EGFR	Epidermal growth factor receptor
SLNs	Solid lipid nanoparticles
NLCs	Nanostructured lipid carriers
AMPK	AMP-activated protein kinase
MMPs	Matrix metalloproteinases
CDs	Carbon dots
PLGA	Poly(lactic-co-glycolic acid)
HPLC	High-performance liquid chromatography
AuNPs	Gold nanoparticles
VEGF	Vascular endothelial growth factor
IONPs	Iron oxide nanoparticles
EMT	Epithelial–mesenchymal transition
NSCLC	Non-small cell lung cancer
XRD	X-ray diffraction
ER-α36	Estrogen receptor alpha 36
TKIs	Tyrosine kinase inhibitors
PAMAM	Polyamidoamine
GMP	Good Manufacturing Practice
CYP	Cytochrome P450
PK–PD	Pharmacokinetic–pharmacodynamic
PEG	Polyethylene glycol
IC50	Half-maximal inhibitory concentration
HIF-1α	Hypoxia-inducible factor 1-alpha
HER2	Human epidermal growth factor receptor 2
USE	Ultrasonic-assisted solvent extraction
EAE	Enzyme-assisted extraction
UPE	Ultra-high pressure extraction
MAE	Microwave-assisted extraction
SFE	Supercritical fluid extraction
ROS	Reactive oxygen species
ABC	ATP-binding cassette
LC-MS/MS	Liquid chromatography–tandem mass spectrometry
Cmax	Maximum plasma concentration
P-gp	P-glycoprotein
TNBC	Triple-negative breast cancer
AUC	Area under the concentration–time curve
